# Rewired cellular signaling coordinates sugar and hypoxic responses for anaerobic xylose fermentation in yeast

**DOI:** 10.1371/journal.pgen.1008037

**Published:** 2019-03-11

**Authors:** Kevin S. Myers, Nicholas M. Riley, Matthew E. MacGilvray, Trey K. Sato, Mick McGee, Justin Heilberger, Joshua J. Coon, Audrey P. Gasch

**Affiliations:** 1 Great Lakes Bioenergy Research Center, University of Wisconsin-Madison, Madison, WI, United States of America; 2 Department of Chemistry, University of Wisconsin-Madison, Madison, WI, United States of America; 3 Laboratory of Genetics, University of Wisconsin-Madison, Madison, WI, United States of America; 4 Genome Center of Wisconsin, University of Wisconsin-Madison, Madison, WI, United States of America; 5 Department of Biomolecular Chemistry, University of Wisconsin-Madison, Madison, WI, United States of America; 6 Morgridge Institute for Research, Madison, WI, United States of America; Pacific Northwest Research Institute, UNITED STATES

## Abstract

Microbes can be metabolically engineered to produce biofuels and biochemicals, but rerouting metabolic flux toward products is a major hurdle without a systems-level understanding of how cellular flux is controlled. To understand flux rerouting, we investigated a panel of *Saccharomyces cerevisiae* strains with progressive improvements in anaerobic fermentation of xylose, a sugar abundant in sustainable plant biomass used for biofuel production. We combined comparative transcriptomics, proteomics, and phosphoproteomics with network analysis to understand the physiology of improved anaerobic xylose fermentation. Our results show that upstream regulatory changes produce a suite of physiological effects that collectively impact the phenotype. Evolved strains show an unusual co-activation of Protein Kinase A (PKA) and Snf1, thus combining responses seen during feast on glucose and famine on non-preferred sugars. Surprisingly, these regulatory changes were required to mount the hypoxic response when cells were grown on xylose, revealing a previously unknown connection between sugar source and anaerobic response. Network analysis identified several downstream transcription factors that play a significant, but on their own minor, role in anaerobic xylose fermentation, consistent with the combinatorial effects of small-impact changes. We also discovered that different routes of PKA activation produce distinct phenotypes: deletion of the RAS/PKA inhibitor *IRA2* promotes xylose growth and metabolism, whereas deletion of PKA inhibitor *BCY1* decouples growth from metabolism to enable robust fermentation without division. Comparing phosphoproteomic changes across *ira2Δ* and *bcy1Δ* strains implicated regulatory changes linked to xylose-dependent growth versus metabolism. Together, our results present a picture of the metabolic logic behind anaerobic xylose flux and suggest that widespread cellular remodeling, rather than individual metabolic changes, is an important goal for metabolic engineering.

## Author summary

An important strategy for sustainable energy is microbial production of biofuels from non-food plant material. However, many microbes, including yeast, cannot use the xylose comprising ~30% of hemicellulosic sugars, especially under anaerobic conditions. Although cells can be engineered with required enzymes, they fail to recognize xylose as a consumable sugar for unknown reasons. We used comparative systems biology across strains with progressive improvements in xylose utilization to understand the metabolic and regulatory logic of anaerobic xylose fermentation. Mutations in evolved strains trigger signaling pathways that are normally antagonistic, producing a cascade of regulatory events coordinating metabolism and growth. Integrative modeling implicates causal events linked to growth versus metabolism and shows the hypoxic response is dependent on carbon sensing in yeast.

## Introduction

Engineering microbes for non-native metabolic capabilities is a major goal in strain engineering. Introducing new genes, gene sets, and now complex pathways [1–3] is relatively facile in modern genetics to imbue strains with novel metabolic capacity. But producing strains that make sufficient quantities of metabolic products remains a major hurdle. The reason likely has less to do with metabolic potential and more to do with how cells regulate activities of enzymes, pathways, and other cellular processes in the context of a cellular system. A better understanding of cellular regulatory systems that can modulate metabolism without producing undesired off-target effects is an active area research for industrial microbiology [4–6].

An example of this is seen in yeast fermentation of non-native sugars present in plant material. Lignocellulosic plant biomass is a renewable substrate for biofuel production, but many microbes cannot natively use the pentoses that comprise a large fraction of the sugars [7,8]. Budding yeast *Saccharomyces cerevisiae* is among the microbes that do not natively recognize xylose as a fermentable sugar, and even when engineered with conversion enzymes strains display low xylose utilization rates [8]. Many studies have attempted to improve xylose metabolism, for example by optimizing xylose metabolism proteins [9–11], mutating or over-expressing xylose transporters [12–14], inducing genes in the pentose-phosphate pathway [15–20], or deleting pathways that siphon intermediates [21–24]. While these modifications improve the phenotype, many of the individual mutations often do so with relatively small effects [12,13,15,17,21–25]. Other studies have used laboratory evolution to select for mutations that enable cell growth on xylose as a sole carbon source, and these approaches have had success [18,21,22,24–27]. But in many cases the reason for improved xylose metabolism remains unknown, which does not advance strategies for rationale engineering.

Here, we dissected the physiology of anaerobic xylose fermentation, studying a previously evolved series of yeast strains we generated [28,29]. Stress-tolerant strain Y22-3 was minimally engineered with xylose isomerase and other genes required for xylose metabolism but was unable to metabolize xylose. This strain was passaged aerobically on xylose-containing medium to produce the Y127 strain that respires xylose aerobically but cannot use xylose anaerobically. Y127 was thus further evolved without oxygen, generating strain Y128 that can ferment xylose to ethanol anaerobically with yields similar to other engineered strains (S1 Table). Null mutations in iron-sulfur cluster scaffold *ISU1* and the stress-activated *HOG1* kinase enable xylose respiration in Y127, while additional loss of xylitol reductase *GRE3* and *IRA2*, an inhibitor of RAS/PKA signaling, facilitate anaerobic xylose fermentation by Y128 [29]. The *IRA2* deletion is interesting, because it is expected to up-regulate RAS and Protein Kinase A (PKA) to promote growth: under optimal conditions, yeast maintain high PKA activity via increased cAMP that inactivates the PKA regulatory subunit Bcy1 [30,31]. The mutations identified in Y128 promote xylose utilization in multiple strain backgrounds [29], and similar mutations were identified in an independent study [27], revealing that they have a generalizable impact on strains with the metabolic potential for xylose consumption. Furthermore, mutations in PKA regulators, including *IRA2*, frequently emerge in laboratory evolution studies that select for improved growth under various conditions [32–39]. Yet the physiological impacts of these mutations that enable improved phenotypes, in particular anaerobic xylose fermentation, remain unclear.

We used comparative multi-omics across the strain panel to distinguish transcript, protein, and phospho-protein differences that correlate with, and in several validated cases cause, improved xylose utilization. Integrating these results presents a systems-view of anaerobic xylose fermentation in yeast, which spans many individual responses that improve the phenotype. Our results support that augmenting cellular signaling to remodel many downstream effects that collectively improve the phenotype underlies the benefits in strain Y128. In the process of this work, we present new insights into Snf1 and PKA signaling and the role of PKA mutations in laboratory evolutions.

## Results

**Fig 1:**
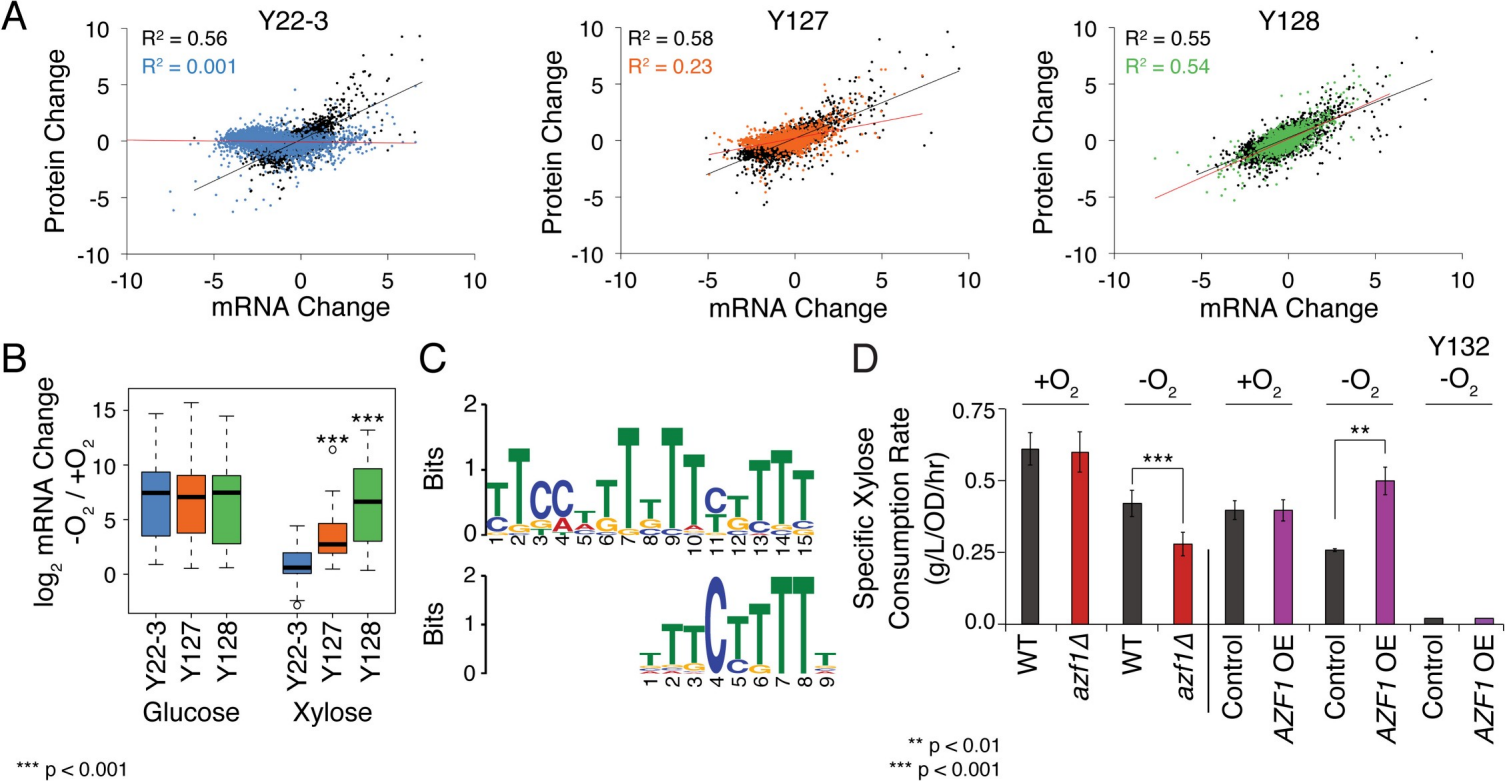
Response to anaerobiosis in xylose-grown cells. A) Log_2_(fold change) in mRNA and protein for cells grown ±O_2_, on glucose (black) or xylose (colored), with linear fit (R^2^) listed. B) Expression of 21 classically defined hypoxic genes (S3 Table). Asterisks indicate significant differences in mRNA change relative to Y22-3 (paired T-test). C) Identified promoter element (top) and known Azf1 site [134] (bottom). D) Average (n = 3) and standard deviation of xylose utilization rates in marker-rescued Y128 (Y133) wild-type (‘WT’) or strains lacking *AZF1* (*azf1Δ*), over-expressing (OE) *AZF1*, or harboring an empty vector (‘Control’) during exponential growth. Xylose utilization rates in marker-rescued Y127 (Y132) empty vector (‘Control’) and OE of *AZF1* are included as indicated. Different growth conditions in the two experiments prevent direct comparison. Asterisks indicate significant differences as indicated (paired T-test).

We first compared the transcriptome and proteome responses of parental strain Y22-3 and evolved strains Y127 and Y128 growing on glucose or xylose, with or without oxygen. For all three strains, glucose-grown cells showed large changes in both mRNA and encoded proteins when shifted to anaerobiosis (Fig 1A). Surprisingly, however, the strains showed major differences when the anaerobic shift was performed on xylose: Y22-3 showed large changes in mRNA but little change in the encoded proteins (Fig 1A and S2 Table). Although this strain retained viability during the experiment, its inability to grow anaerobically could have caused a defect in protein production despite major mRNA induction upon the shift. In contrast, strain Y127 was unable to grow yet produced large mRNA changes and moderate changes in the corresponding proteins (S1A Fig). This included several proteins (*e*.*g*. Pdr11 and Anb1) that are not expressed under aerobic conditions but were detected after the anaerobic shift, implicating nascent translation. Nonetheless, the protein changes in xylose-grown Y127 were much smaller than glucose-grown cells shifted to anaerobiosis. In contrast, the correlation between mRNA and protein change was fully recovered in xylose-grown Y128 shifted to anaerobic growth, on par with the correlation seen in glucose-grown cells (Fig 1A). Thus, strain Y127 and especially Y22-3 may have a defect in proteome remodeling during anaerobiosis despite large changes in mRNA, specifically when grown on xylose.

Analysis of the transcriptome data revealed that Y22-3 and to some extent Y127 showed defective induction of *ANB1*, a canonical gene in the hypoxic response that is essential for anaerobic translation [40], whereas Y128 showed robust induction of *ANB1* (S1B Fig). While it remains unclear if defective *ANB1* induction causes the defect in Y22-3 and Y127 protein accumulation, this observation led us to discover that over 70% of genes classically involved in the hypoxic response (S3 Table) were induced at the transcript level in all strains grown on glucose but largely uninduced in xylose-grown Y22-3 and induced progressively stronger in Y127 and Y128, respectively (Fig 1B). Once again, the differences did not correlate perfectly with growth, since Y127 showed a partial response despite no growth. Furthermore, the defect did not correlate with a lack of transcriptional response, since many other transcripts increased in Y22-3 and Y127 shifted to anaerobic xylose conditions (Fig 1A). Instead, this defect reveals a previously unrecognized connection between the hypoxic response and carbon source in yeast.

### Sugar- and oxygen-responsive transcription factors Azf1 and Mga2 influence anaerobic xylose fermentation

To further investigate this effect, we identified 128 transcripts that were induced progressively stronger across the strain panel when shifted to anaerobic-xylose conditions, with a pattern similar to the hypoxic response (see Materials and Methods, S1C Fig and S4 Table). These were enriched for genes involved in the hypoxic response, ergosterol biosynthesis, cysteine metabolism, and translation (p < 1x10^-4^, hypergeometric test). Promoter analysis identified tandem binding sites of Azf1, a transcription factor (TF) responsive to non-preferred sugars [41–43] (Fig 1C). Over half (68) of the 128 progressively induced genes harbored upstream Azf1 motifs (p = 5.7x10^-45^, hypergeometric test), including nearly all of the classical hypoxic genes. Indeed, over-expression of *AZF1* increased rates of growth, xylose consumption, and ethanol production in Y128 –but only when cells were grown on xylose and anaerobically (Figs 1D and S2). In contrast, deletion of *AZF1* decreased growth and sugar fermentation, largely specific to anaerobic xylose growth (Figs 1D and S2). Although statistically significant, it is notable that the impact of *AZF1* was subtle, indicating that it cannot fully explain the improvements in Y128. Furthermore, the effect required Y128 mutations, as it was observed in a different strain background recapitulating Y128 alleles but not in Y22-3 (S3 Fig).

**Fig 2:**
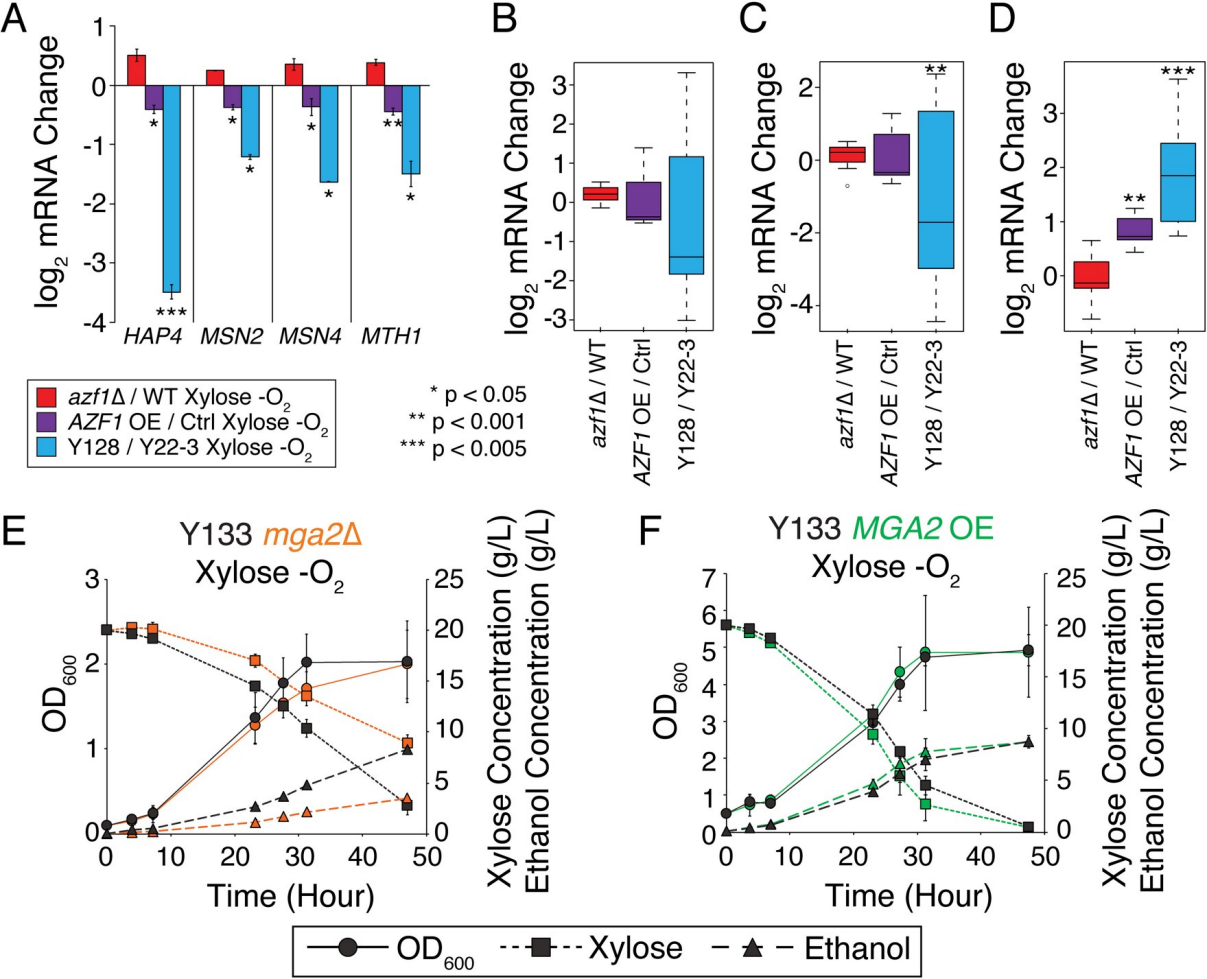
Azf1 and Mga2 regulate anaerobic xylose responses. A) Average log_2_(fold change) in mRNA abundance of denoted genes as listed in the key. B-D) Distributions of log_2_(fold change) in mRNA abundances for Hap4 (B), Msn2/Msn4 (C), and Mga2 (D) targets that are affected by *AZF1* overexpression and show a corresponding change in Y128 versus controls (see text). Asterisks indicate significant difference compared to *azf1Δ* versus WT comparison (paired T-test). E-F) OD_600_ (circles), xylose concentration (squares), and ethanol concentration (triangles) for strain (orange) Y133 (marker-rescued Y128) lacking (*mga2*Δ, orange plot on the left) or over-expressing (‘OE’, green plot on the right) *MGA2*, and Y133 wild type (‘WT’) or empty-vector control (black) during anaerobic growth on xylose. Different growth conditions in the two experiments prevent direct comparison.

We therefore identified transcriptome effects of *AZF1* deletion or over-expression. *AZF1* over-expression in particular had broad effects on the anaerobic-xylose transcriptome, affecting 411 genes (FDR < 0.05) whose expression change also paralleled differences in Y128 compared to Y22-3 (S4A Fig and S5 Table). These were enriched for genes with upstream Azf1 promoter motifs, as expected (p = 3x10^-2^, hypergeometric test). Also, among the affected genes were several TFs and their targets. For example, *AZF1* over-production reduced expression of *HAP4* that regulates respiration genes [44] and *MSN2/MSN4* that induce stress-defense genes [45], and targets of Hap4 (p = 1x10^-3^, hypergeometric test) and Msn2/Msn4 (p = 1x10^-20^) were enriched among genes repressed upon *AZF1* induction (Fig 2A–2C). This was interesting because deletion of *HAP4* and *MSN4* were previously shown to improve xylose consumption [46]. *AZF1* also reduced expression *MTH1*, encoding a repressor of hexose/xylose transporters [47] (Fig 2A), and several sugar transporters that can import xylose were correspondingly induced (S5 Table).

Genes induced upon *AZF1* over-production were also enriched for targets of Mga2 (p = 2x10^-3^), a hypoxia-responsive TF that regulates genes involved in sterol and fatty acid metabolism [48] (Fig 2D)–this was intriguing given that defects in the hypoxic response led us to Azf1 in the first place. To test its importance in anaerobic xylose consumption, we perturbed *MGA2* expression directly. Indeed, *MGA2* deletion or over-expression had subtle but opposing effects on anaerobic xylose (but not glucose) utilization (Fig 2E and 2F). These results show that the sugar-responsive Azf1 and the oxygen-responsive Mga2 play important, but subtle, roles in mediating anaerobic xylose fermentation in Y128.

### Network inference implicates PKA and Snf1 regulatory cascades

We were especially interested in the upstream regulatory network that mediates the downstream response, including activation of Azf1 and Mga2 targets. We therefore profiled the phosphoproteomes of Y22-3, Y127, and Y128 cultured on xylose, with or without oxygen (S6 Table), and applied a novel network approach [49] to infer regulation of strain-specific phosphorylation differences (see Materials and Methods). Because many kinases recognize specific sequences around the phosphorylation site, we identified ‘modules’ of phospho-peptides that are likely co-regulated and then implicated kinases and phosphatases that may control their phosphorylation change. First, we grouped peptides based on their changes in phosphorylation when each strain was shifted from aerobic to anaerobic xylose conditions, identifying peptides with progressive increases or decreases in phosphorylation response across the strain panel (“Class A” increases or decreases) and peptides with responses uniquely higher or lower in Y128 (“Class B” increases or decreases). Next, we partitioned each group into ‘modules’ of peptides that harbor similar sequences around the phosphorylated site (‘phospho-motifs’, see Materials and Methods). Module peptides therefore share the same phosphorylation pattern and similar phospho-motifs, and thus are enriched for peptides that are likely co-regulated [49]. Reasoning that module peptides are regulated by the same upstream regulator(s), we then searched a background network of protein interactions for proteins that physically interact with more module peptides than expected by chance (FDR < 0.05, see Materials and Methods). We focused on kinases whose phosphorylation preference matches the module phospho-motif, thereby implicating those kinases as direct regulators of module peptides.

**Fig 3:**
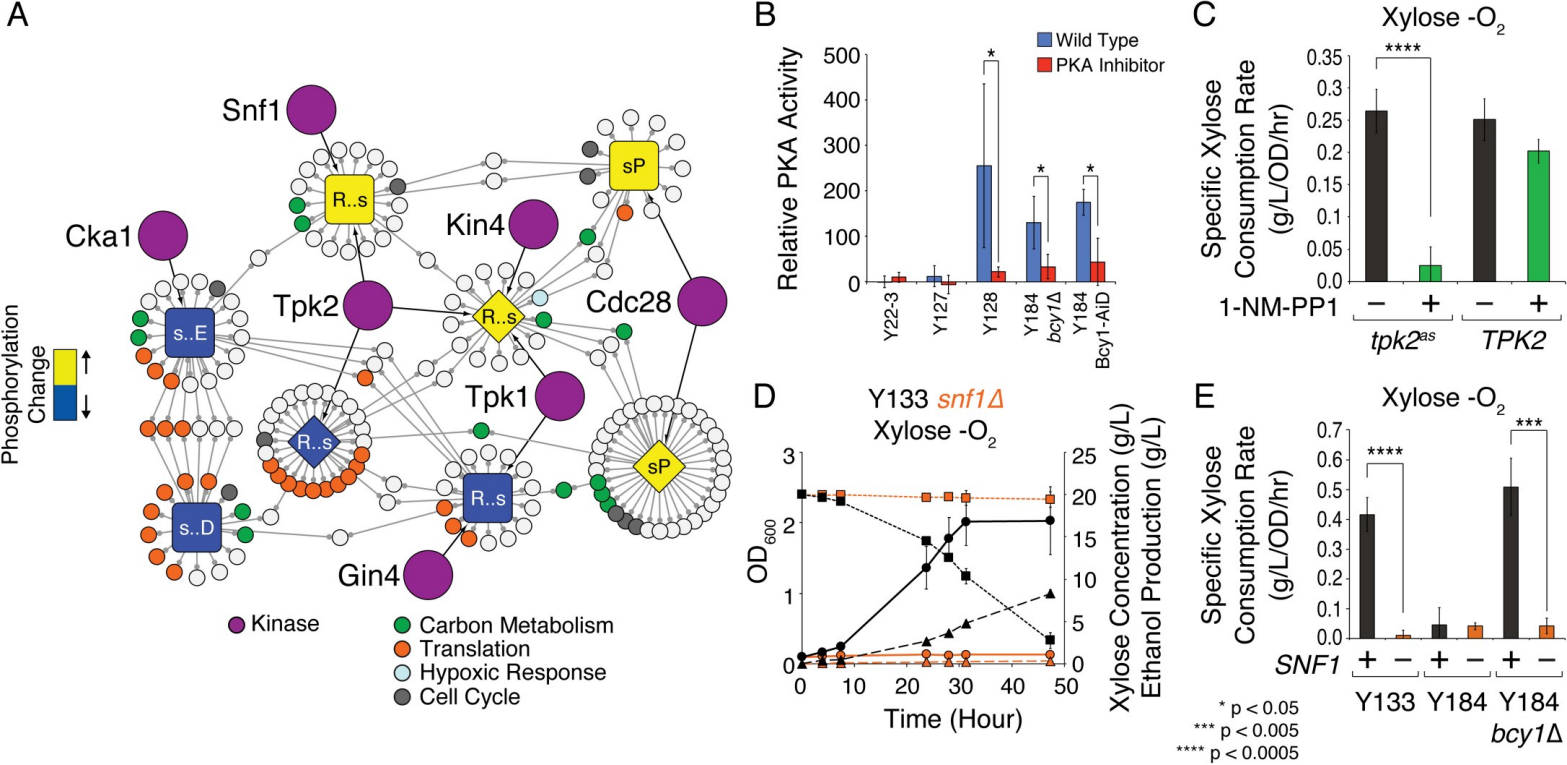
Inferred network regulating phosphorylation changes during anaerobic xylose growth. A) Modules of peptides are shaped and colored according to class (Class A, diamond; Class B, square) and increase (yellow) or decrease (blue) of phosphorylation change across the strain panel, as described in the text. Each module is labeled with the phospho-motif sequence, with small case letter representing the phosphorylated site and “‥” indicating non-specific residues. Implicated kinase regulators are shown as purple circles; proteins whose peptides belong to each module are shown as smaller circles, color-coded by protein function as listed in the key. Note that proteins with multiple phospho-sites can belong to multiple modules. B) Average (n = 3) and standard deviation of the relative *in vitro* phosphorylation of a PKA substrate (ABCAM kit, see Methods) for lysates from cells that can (Y128, Y184 *bcy1Δ*, Y184 Bcy1-AiD–described in text) or cannot (Y22-3, Y127) use xylose anaerobically. Orange bars represent phosphorylation in the presence of PKA inhibitor H-89. C) Average (n = 3) and standard deviation of sugar utilization rates for Y133 *tpk1Δtpk3Δtpk2*^*as*^ or Y133 *tpk1Δtpk3ΔTPK2* during anaerobic growth, in the presence (green) or absence (black) of 1-NM-PP1. D) OD_600_ (circles), xylose concentration (squares), and ethanol concentration (triangles) for WT (black) or *snf1Δ* (orange) Y133 (marker-rescued Y128) grown in xylose -O_2_. E) Average (n = 3) and standard deviation of xylose utilization rates for strains in the presence (+) or absence (-) of *SNF1*. Asterisks indicate significant differences according to the key (paired T-tests).

The resulting network implicated several regulators in the anaerobic xylose response (Fig 3A). Peptides that showed highest phosphorylation levels in Y22-3 upon anaerobic xylose shift included ribosomal proteins and translation factors, whose modules were associated with PKA subunit Tpk2 and Cka1 of the CK2 kinase that phosphorylates translation factors in other organisms to modulate translation [50–52]. Other modules showed increased phosphorylation in Y128, including those connected to cyclin-dependent kinase Cdc28 that regulates carbon-metabolism enzymes [53,54] and proteins required for division.

We were intrigued by multiple modules connected to PKA subunits Tpk1 and Tpk2, since mutations in *IRA2* are predicted to up-regulate RAS/PKA signaling [29,55,56]. Two PKA-associated modules showed reduced phosphorylation in Y128, spanning translation factors described above–indeed, the proteins whose peptides belong to these two modules are enriched for known targets of PKA (p = 3x10^-3^), implicating the other peptides as potential PKA substrates [49]. But two other modules of peptides showed increased phosphorylation in xylose-grown Y128 shifted to anaerobic conditions (Fig 3A). These modules included known PKA targets and phospho-sites (S5 Fig), such as hexokinase 2 that promotes glycolytic flux and stress-responsive TF Msn2 that is inhibited by PKA phosphorylation [57]. Intriguingly, this module also included hypoxia-responsive Mga2 at a site that matches PKA specificity [48]. *MGA2* genetically interacts with *IRA2* in high-throughput datasets [58], further supporting a link between PKA and *MGA2* function, and Mga2 targets are up-regulated in Y128 (S4B Fig). Together, these results suggest that signaling through PKA is modulated in Y128.

Indeed, further experiments verified the importance of PKA signaling. First, lysate from anaerobic-xylose grown Y128 showed increased phosphorylation of a PKA substrate *in vitro*, which was blocked by PKA inhibitor H-89 (Fig 3B). Second, Y128 harboring a single analog-sensitive allele of PKA subunits (*tpk2*^*as*^) required PKA function for anaerobic xylose consumption. Inhibition of *tpk2*^as^ with analog 1-NM-PP1 rapidly inhibited growth and anaerobic xylose fermentation (Figs 3C and S6A–S6C). We found no difference in the role for different PKA subunits (S6D Fig). Third, the beneficial effects of *AZF1* over-expression required deletion of *IRA2* (S6E and S6F Fig). Together, these results show that increased RAS/PKA activity is required for anaerobic xylose fermentation in Y128, even though some proteins known to be regulated by PKA show decreased phosphorylation in these conditions (S5B Fig and below).

One of the Tpk2-connected modules was also associated with the Snf1 kinase, which is activated by non-preferred carbon sources to induce alternative-carbon utilization genes [59–62]. This was interesting, because PKA and Snf1 are not normally highly active under the same conditions–the two regulators can produce antagonistic effects and even inhibit each other’s activity [30,31,55,62]. To test this network prediction, we knocked out *SNF1* from marker-rescued Y128 (strain Y133) and measured xylose fermentation capabilities. Indeed, *SNF1* is essential for anaerobic xylose utilization in Y133, although it is insufficient in the absence of PKA-activating mutations (Figs 3D and 3E and S7). Surprisingly, Snf1 was also essential for anaerobic growth on glucose when *IRA2* was deleted (S7 Fig), indicating a previously unknown role for Snf1 and PKA in oxygen responses (see Discussion). Thus, both increased PKA activity and *SNF1* are required for anaerobic xylose fermentation, validating the network predictions.

### Deletion of PKA regulator BCY1 decouples growth from metabolism

**Fig 4:**
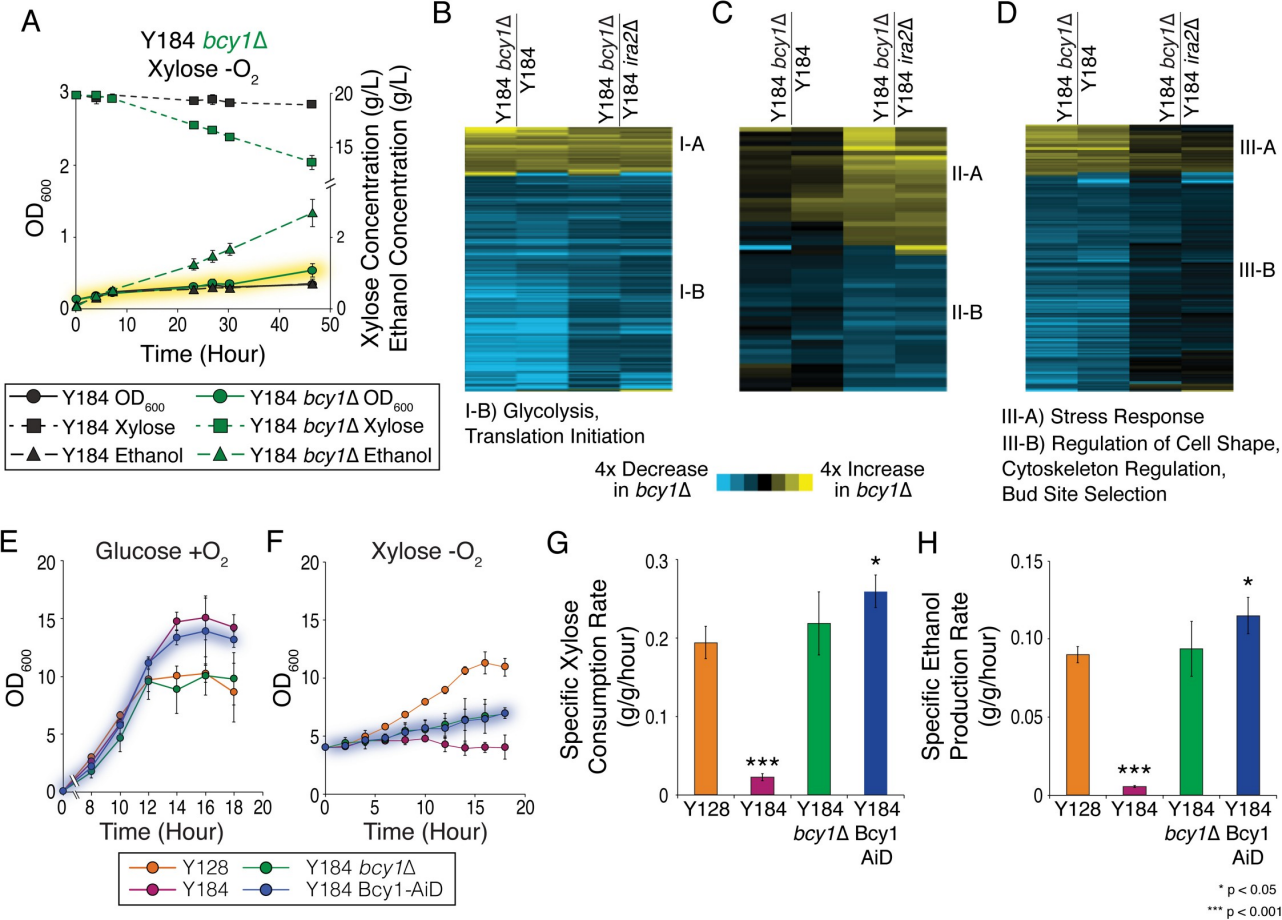
Mutation of *BCY1* decouples growth from anaerobic xylose metabolism. A) OD_600_ (circles), xylose concentration (squares), and ethanol concentration (triangles) for Y184 (Y22-3 *gre3Δisu1Δ*) (black) and Y184 *bcy1Δ* (green) during anaerobic growth on xylose. Note that the culture was inoculated at low OD to show the effect; cells do not use all the xylose because there are very few cells in the experiment. B-D) Phospho-peptide changes in Y184 *bcy1Δ* relative to references, for phospho-peptides (rows) specific to Y184 *bcy1Δ* (B) or similar to Y184 (C) or Y184 *ira2Δ* (D). Functional enrichments for each denoted cluster are listed below each heat map. (E-F) Growth of strains in glucose +O_2_ (E) and xylose -O_2_ (F) as indicated in the key. (G-H) Average (n = 3) specific xylose consumption rate (G) or ethanol production rate (H). Asterisks indicate significant differences relative to Y128 (paired T-test).

Deletion of *IRA2* upregulates multiple downstream effects of RAS, including PKA activation [63–65]. To distinguish if PKA induction is sufficient for the response, we deleted the PKA negative regulatory subunit *BCY1* in strain Y184 (Y22-3 *gre3Δ isu1Δ*) that can use xylose aerobically but not anaerobically. If PKA up-regulation is sufficient, then *BCY1* deletion should enable anaerobic growth and metabolism of xylose similar to when *IRA2* is deleted. However, this was not the case: Y184 lacking *BCY1* could not grow anaerobically on xylose, as known for *bcy1Δ* strains on other non-preferred carbon sources [66]–but surprisingly the cells fermented xylose despite growth arrest, at ethanol yields (~0.45 g/g xylose) matching or surpassing other published xylose-converting strains (Figs 4A and S8 and S1 Table). Lysate from anaerobic xylose-grown Y184 *bcy1Δ* showed increased PKA activity *in vitro* that was blocked by the H-89 PKA inhibitor (Fig 3B). Thus, up-regulating PKA activity through *BCY1* deletion enabled xylose fermentation but in the absence of growth.

### Dissecting phosphorylation events linked to anaerobic xylose metabolism versus growth

The unique phenotype of the Y184 *bcy1Δ* strain provided an opportunity to distinguish phosphorylation events correlated with growth *versus* metabolism. Phosphorylation patterns shared between Y184 and Y184 *bcy1Δ*, neither of which can grow anaerobically on xylose, are therefore associated with growth arrest; in contrast, phosphorylation patterns common to Y184 *bcy1Δ* and Y184 *ira2Δ*, which share the ability to ferment xylose anaerobically but differ in growth capabilities, are implicated in xylose metabolism (Fig 4B–4D and S7 Table and S1 Text). The 210 peptides whose phosphorylation levels were unique to Y184 *bcy1Δ* or shared between non-growing strains occurred on proteins involved in translation, ribosome biogenesis, nucleotide biosynthesis (including ribonucleotide reductase Rnr2), and DNA replication–all functions required for division. Many of these phosphorylation patterns are likely an indirect consequence of arrest. To test if growth arrest via direct inhibition of Rnr2 could block growth but enable fermentation, we used the RNR inhibitor hydroxyurea to arrest growth; but this treatment also halted xylose utilization (S9 Fig).

In contrast, many of the 335 phosphorylation patterns unique to Y184 *bcy1Δ* or shared between the xylose-fermenting strains were linked to metabolism, including on hexose transporters Hxt2 and Hxt6 that are already known to influence xylose uptake [67,68], enzymes involved in glycolysis (Pfk2, Fbp26, Tdh1/2, Cdc19, Pda1, Pdc1), trehalose biosynthesis that regulates glycolytic overflow (Tsl1, Tps2, Tps3, Nth2), and glycerol and alcohol dehydrogenases that recycle NADH during high glycolytic flux (Gpd1, Gut1, Adh1). Augmentation of proteins in these pathways have been implicated in improved xylose utilization [69–74].

Several phosphorylation patterns implicated in Y128 (Fig 3A) were not recapitulated in the Y184 *bcy1Δ* strain, suggesting that they are not strictly required for anaerobic xylose fermentation. For example, unlike Y128, phosphorylation of known Cdc28 targets was reduced in Y184 *bcy1*Δ compared to Y184 *ira2Δ*, strongly suggesting that Cdc28-dependent phosphorylation in Y128 is linked to division and not xylose metabolism. Despite increased PKA signaling in the *bcy1Δ* strain (Fig 3B), several of the known and predicted PKA phosphorylation sites in Y128 showed reduced phosphorylation upon *BCY1* deletion. For example, relative to Y128, Y184 *bcy1Δ* showed decreased phosphorylation of serine 15 (S15) on the main hexokinase, Hxk2, whose phosphorylation normally increases activity [75]. Finally, the Y184 *bcy1Δ* strain displayed several unique phosphorylation patterns not observed in the other strains. Remarkably, this included decreased phosphorylation on Hog1 activating site T174, seen when Hog1 activity is reduced [76]. This suggests that effects of *BCY1* deletion mimic Hog1 inactivation that enhances xylose consumption [27,29], and raises the possibility that PKA activity can suppress Hog1 activation.

### Perturbing Bcy1 sequence through protein fusion recapitulates the *bcy1*Δ phenotype

Although *BCY1* deletion enhances anaerobic xylose metabolism, it slows aerobic growth on glucose [66], which is a problem for industrial propagation of microbial cells. As a proof-of-principle for industrial use, we therefore generated a tagged version of Bcy1 in an attempt to enable auxin-dependent degradation [77] and made an important discovery: simply fusing a peptide to the carboxyl-terminus of Bcy1 (without enabling degradation) was enough to combine the benefits of *BCY1+* and *bcy1Δ* strains in aerobic and anaerobic conditions, respectively (Fig 4E–4H, see Materials and Methods). When grown aerobically on glucose to mimic industrial propagation, cells expressing a Bcy1-AiD fusion (but without auxin-regulated controllers) grew to higher cell titers than Y128, consistent with functional Bcy1 activity (Fig 4E). But when shifted at high density to anaerobic xylose conditions, the strain dramatically reduced growth and robustly fermented xylose to ethanol with high yield (~0.45 g/g xylose), mimicking the *bcy1Δ* strain (Fig 4F–4H and S1 Table). The Bcy1 protein fusion remained readily detectible by Western blot after anaerobic shift, indicating that recapitulation of the *bcy1Δ* phenotype was not through Bcy1 degradation (S10 Fig). These results and our network analysis raise the possibility of more subtle modulation of PKA activity then simple up-regulation (see Discussion).

## Discussion

**Fig 5:**
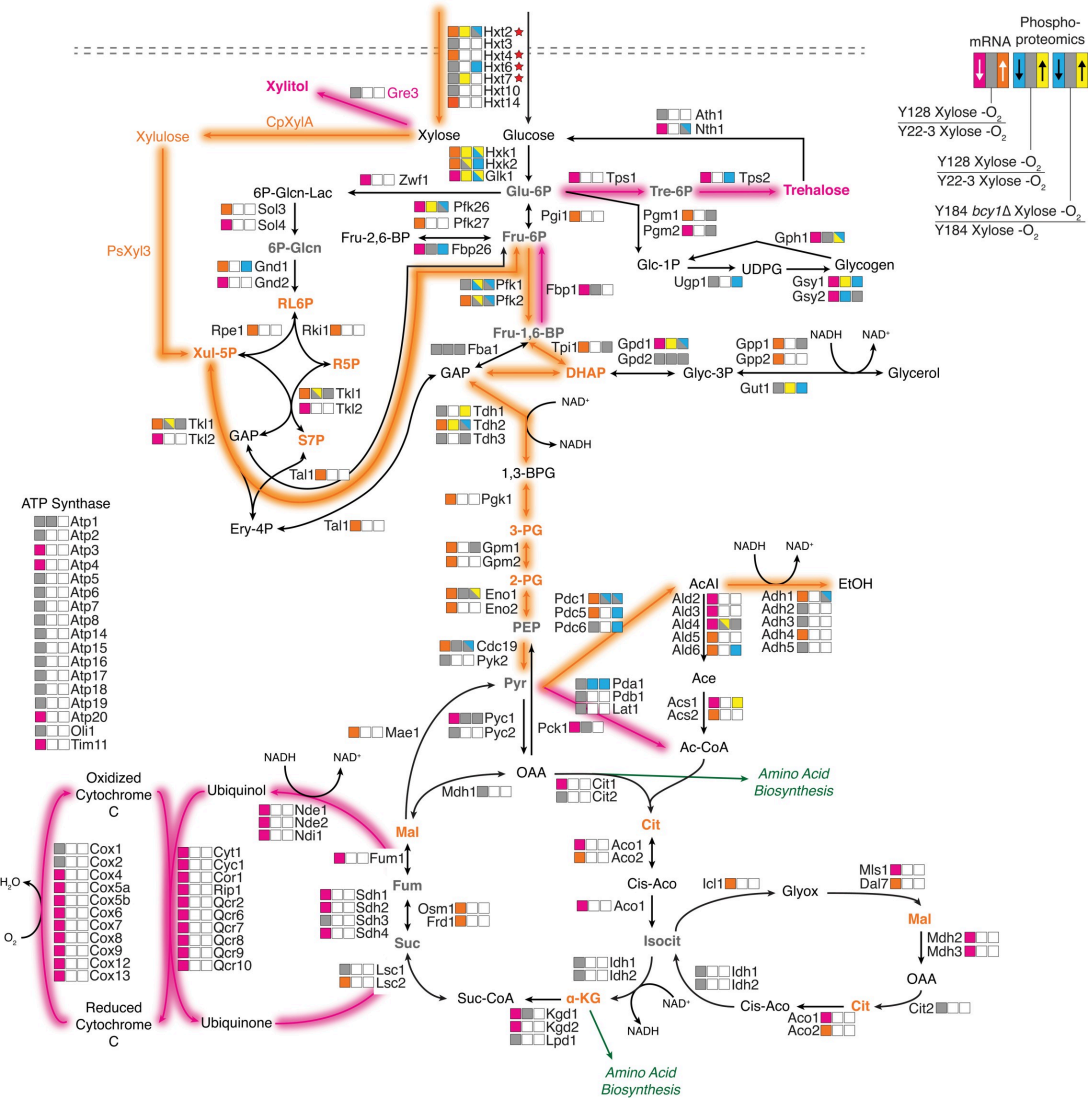
Integrative model incorporating transcript, phospho-protein, and metabolite changes across the strain panel. Map of central carbon metabolism. Each step is annotated with boxes indicating mRNA difference (left) or phosphorylation difference (middle) in Y128 versus Y22-3, or phosphorylation difference (right) in Y184 *bcy1Δ* versus Y184 grown anaerobically on xylose, according to the key. Gray indicates no significant change, white represents missing data, and multi-colored blue/yellow boxes indicate multiple phospho-sites with different changes. Metabolites measured previously [29] are colored to indicate an increase (orange) or decrease (magenta) in abundance in Y128 versus Y22-3 grown anaerobically on xylose. Reactions predicted to be active (orange) or suppressed (magenta) in xylose fermenting strains based on mRNA, protein, and/or metabolite abundances are highlighted. Hexose transporters marked with a star have been implicated in xylose transport.

Our results provide new insight into the upstream regulatory network that enables anaerobic xylose fermentation and the downstream cellular responses that mediate it. Evolved strain Y128 activates PKA signaling while requiring Snf1, leading to a cascade of downstream effects that involve the sugar-responsive Azf1, oxygen-responsive Mga2, and downstream effectors that control respiration (Hap4), stress response (Msn2/Msn4), and sugar transport (Mth1) among others. Integrating transcriptomic, phosphoproteomic, and metabolomic data [29] across the strain panel provides a glimpse of the downstream cellular response (Fig 5), with combined effects including induction of sugar transporters, up-regulation of genes and metabolites in the non-oxidative branch of the pentose phosphate pathway, increased abundance of xylolytic and glycolytic intermediates, reduced abundance of overflow metabolites, and sharp reduction in respiration components. Our results support previous metabolic engineering studies that suggested the need for widespread cellular remodeling, in addition to individual metabolic changes, for optimal product production [78–83].

Many of the downstream responses we identified have been implicated before in improved xylose fermentation, including over-expression of xylose transporters, pentose-phosphate pathway enzymes, modulation of hexokinase, and down-regulated stress-activated transcription factors [12–18,21–26,72,84–87]. However, in many prior studies, alteration of individual genes through deletion or over-expression often has only minor effects on the phenotype [12,13,15,17,21–25]. This is also consistent with our results: although we validated predictions to show that Azf1 and Mga2 contribute to anaerobic xylose fermentation, their individual effects are small compared to the impact of upstream regulatory changes. Thus, the improvement seen in Y128 compared to its progenitors emerges from the combined effects of many downstream changes that collectively impact the phenotype. This is also consistent with the literature showing that combinatorial gene mutation produces additive or epistatic effects [12,25,27,88,89]. For example, Papapetridis *et al*. [25] tested the effects of individual gene deletions on co-fermentation of glucose and xylose; the most significant benefits emerge when multiple genes were combined, *e*.*g*. co-deletion of hexokinase *HXK2* and ubiquitin ligase *RSP5*. Over-expression of transaldolase (*TAL1*) or glyceraldehyde-3-phosphate dehydrogenase (*GDP1*), or deletion of phosphatase *PHO13* or glucose-6-phosphate dehydrogenase (*ZWF1*), all impact xylose fermentation individually, but combinatorial effects emerge from combining *TAL1* induction and *PHO13* deletion [89] or *GPD1* over-expression with *ZWF1* ablation [88]. Predicting which combinatorial modifications to make is likely to be a significant challenge going forward, especially for mutations outside of known metabolic pathways. It is in this light that selecting for regulatory changes through laboratory evolution studies can produce combinatorial effects of large combined impact.

The benefits in Y128 emerge in part through co-activation of PKA along with Snf1. Snf1 is required for growth on non-preferred carbon sources, and thus its involvement in xylose utilization does not seem surprising [90]. However, Snf1 alone is not sufficient to enable anaerobic xylose fermentation unless *IRA2* or *BCY1* are also deleted (Fig 3D and 3E). The response of strain Y128 combines those normally seen on poor carbon sources (*i*.*e*. induced expression of hexose/xylose transporters, altered hexokinase regulation, and Azf1 activation) and those typically seen on abundant glucose (*i*.*e*. phosphorylation events associated with increased glycolytic flux, reduced expression of respiration and stress-responsive genes, and active PKA signaling). Surprisingly, Snf1 is specifically required for anaerobic growth, on both xylose and glucose, in the context of Y128 mutations. The link of Snf1 to anaerobic growth has not been reported to our knowledge, although Snf1 has recently been tied to oxygen responses: glucose-grown yeast exposed to hypoxia phase-separate glycolytic enzymes in a Snf1-dependent manner, in a process influenced by Ira2 [91]. Snf1 and PKA are not normally coactivated in yeast [30,31,62], with the primary exception of invasive growth, a foraging response in which starved cells invade a solid substrate [92,93]. This ecological response may explain the link between sugar and oxygen responses, since cells undergoing substrate invasion may prepare for impending hypoxia.

Numerous laboratory evolution studies selecting for improved growth identified mutations in PKA signaling, including in strains evolved under sugar and nitrogen limitation, continuous growth in rich medium, growth on non-preferred carbon sources, and on industrial wort [32–34,36,38,39]. These studies often identify mutations in RAS and RAS regulators, adenylate cyclase that generates cAMP, and especially *IRA2*. *IRA2* mutations have also been implicated as frequent second site suppressors of other mutations to enable improved growth [37]. Yet mutations in *BCY1* are not generally identified in laboratory evolutions. This may reveal an important link: that while up-regulating RAS improves growth, which is the primary selection in most laboratory evolution studies, up-regulating PKA by Bcy1 deletion can have important effects including on industrially relevant traits that could be missed by growth-based assays. It will be important to dissect the different physiological effects of increased PKA activity via RAS up-regulation (*i*.*e*. *IRA2* deletion) or PKA modulation directly via *BCY1* deletion.

At the same time, our results suggest that PKA is not simply up-regulated, but rather that cellular signaling is ‘rewired’ to up-regulate some PKA targets while disfavoring others. This could emerge from differential activation of phosphatases, but could also occur if PKA is being directed to different sets of targets (perhaps through differential composition of PKA via the three Tpk subunits [94–97]). Bcy1 is thought to direct PKA to specific proteins, much like AKAP proteins in mammals [98,99]. Our results that a Bcy1 peptide fusion (Bcy1-AiD) combines the benefits of BCY1+ cells in aerobic glucose medium and *bcy1Δ* cells in anaerobic xylose suggest a complex interplay. Recent studies in mammalian cells reveal that the PKA regulatory subunit does not disassociate from catalytic PKA subunits upon cAMP binding [100], raising the possibility that structural differences in Bcy1-AiD could direct PKA to different sets of proteins. That some well-characterized PKA phospho-sites are up-regulated while others are suppressed in anaerobically-grown Y128 supports this hypothesis. Future work will be required to elucidate the direct molecular connections. Furthermore, how these changes impact other traits important to industrial conditions–including stresses of complex lignocellulosic plant material, high mixed sugar concentrations, and alternate nutrient availability, will be important considerations.

## Materials and methods

### Media and growth conditions

Cells were grown in YP medium (10 g/L yeast extract, 20 g/L peptone) with glucose or xylose added at 20 g/L final concentration, unless otherwise noted. Antibiotics were added where indicated at the following concentrations: 200 mg/L G418, 300 mg/L Hygromycin B, 100 mg/L ClonNat. For aerobic growth, cultures were grown at 30°C with vigorous shaking in flasks. For anaerobic growth, media was incubated at 30°C in a Coy anaerobic chamber (10% CO_2_, 10% H_2_, and 80% N_2_) for ≥16 hours before inoculation, and cultures were grown at 30°C in flasks using stir bars spinning at 300 rpm to reduce flocculation. Cultures were inoculated with a saturated culture of cells grown aerobically in YP-glucose medium, washed one time with the desired growth media, at the specified OD_600_ value as indicated. Cell growth was measured using OD_600_, and extracellular sugar and ethanol concentrations were measured with HPLC-RID (Refractive Index Detector) analysis [28].

### Strains and cloning

*Saccharomyces cerevisiae* strains used in this study are described in Table 1. The creation of Y22-3, Y127, and Y128 and their antibiotic marker-rescued counterparts with the *KanMX* gene removed (Y36, Y132, and Y133, respectively) was described previously [29]. All strains express the minimal required genes for xylose metabolism, including xylose isomerase (*xylA* from *Clostridium phytofermentans*), xylulose kinase (*XYL3* from *Scheffersomyces stipites*), and transaldolase (*TAL3* from *S*. *cerevisiae*). Gene knockouts were generated by homologous recombination of the *KanMX* or *Hph* cassettes into the locus of interest and verified using multiple diagnostic PCRs. *AZF1* and *MGA2* were over-expressed using the MoBY 2.0 plasmid and empty vector as a control [101], growing cells in medium containing G418 to maintain the plasmid. *BCY1* was deleted from indicated strains through homologous recombination of the *KanMX* cassette and verified by multiple diagnostic PCRs. Strain Y184 harboring integrated *BCY1-AiD* [77,102–104] (Auxin-induced-Degron) was generated as follows: all plasmids were provided by National BioResource Program (NBRP) of the Ministry of Education, Culture, Sports and Technology (MEXT), Japan. Plasmid pST1933 (NBRP ID BYP8880) [103] containing 3x Mini-AiD sequences, 5x FLAG Tag and *KanMX* was modified to include the 329 bp of *BCY1* 3´ UTR between the 5x FLAG tag and the *KanMX* marker gene. This construct (3x Mini-AiD, 5x FLAG tag, *BCY1* 3´ UTR, and *KanMX*) was amplified and inserted downstream and in-frame of *BCY1* in Y184 (Y22-3 *gre3Δisu1Δ*) to form strain Y184 Bcy1-AiD. The integrated construct was verified by sequencing. Neither the pTIR plasmid enabling auxin-depedent degradation nor auxin was required for the desired effect (not shown), thus these were omitted from the analysis. Phenotypes introduced by *BCY1* deletion were complemented by introducing *BCY1* on a CEN plasmid: to generate the plasmid, *BCY1* and 1000 bp upstream and 1000 bp downstream were amplified from Y128 and inserted into a *NatMX*-marked CEN plasmid via homologous recombination and sequence verified. This plasmid or the empty vector (pEMPTY) were transformed into appropriate strains. Phenotypes resulting from *SNF1* deletion were complemented using the *SNF1* MoBY 2.0 plasmid [101] and compared to the empty vector control.

**Table 1:** Strains used in this study.

Strain Name	Description	Ref
Y22-3	CRB Strain with xylose utilization genes (G418^R^)	[28]
Y127	Evolved Y22-3 for aerobic xylose utilization (G418^R^)	[28]
Y128	Evolved Y127 for anaerobic xylose utilization (G418^R^)	[28]
Y36	Y22-3 marker-rescued (MR)—lacking *KanMX* cassette	[28]
Y132	Y127 marker-rescued (MR)—lacking *KanMX* cassette	[28]
Y133	Y128 marker-rescued (MR)—lacking *KanMX* cassette	[28]
Y133 *azf1Δ*	Y133 *azf1Δ*::*KanMX* (G418^R^)	This Study
Y133 *AZF1* MoBY	Y133 containing *AZF1* MoBY 2.0 Plasmid (G418^R^)	This Study
Y133 MoBY Control	Y133 containing Empty Vector MoBY 2.0 Plasmid (G418^R^)	This Study
Y36 *AZF1* MoBY	Y36 containing *AZF1* MoBY 2.0 Plasmid (G418^R^)	This Study
Y36 MoBY Control	Y36 containing Empty Vector MoBY 2.0 Plasmid (G418^R^)	This Study
CEN.PK113-5D Xylose Strain	CEN.PK113-5D with *HOΔ*::*ScTAL1-CpxylA-SsXYL3-loxP*, *isu1Δ*::*loxP*, *hog1Δ*::*kanMX*, *gre3Δ*::*loxP*, *ira2Δ*::*loxP*	[29]
CEN.PK113-5D Xylose Strain *AZF1* MoBY	CEN.PK113-5D Xylose Strain containing *AZF1*-MoBY 2.0 Plasmid (G418^R^)	This Study
CEN.PK113-5D Xylose Strain MoBY Control	CEN.PK113-5D Xylose Strain containing Empty Vector MoBY 2.0 Plasmid (G418^R^)	This Study
Y184	Y22-3 *gre3*Δ::MR *isu1*Δ::loxP-Hyg (Hyg^R^)	This Study
Y243	Y22-3 *gre3*Δ::MR *isu1*Δ::loxP-Hyg *ira2*Δ::MR (Hyg^R^)	[29]
Y132 *bcy1Δ*	Y132 *bcy1Δ*::*KanMX* (G418^R^)	This Study
Y184 *bcy1Δ*	Y22-3 *gre3*Δ::MR *isu1*Δ::loxP-Hyg *bcy1Δ*::*KanMX* (Hyg^R^, G418^R^)	This Study
Y243 *bcy1Δ*	Y22-3 *gre3*Δ::MR *isu1*Δ::loxP-Hyg *ira2*Δ::MR *bcy1Δ*::*KanMX* (Hyg^R^, G418^R^)	This Study
Y133 *snf1*Δ	Y133 *snf1Δ*::*Hyg* (Hyg^R^)	This Study
Y184 *snf1*Δ	Y22-3 *gre3*Δ::MR *isu1*Δ::loxP-Hyg *snf1Δ*::*NatMX* (Hyg^R^, Nat^R^)	This Study
Y184 *bcy1*Δ*snf1*Δ	Y22-3 *gre3*Δ::MR *isu1*Δ::loxP-Hyg *bcy1Δ*::*KanMX snf1Δ*::*NatMX* (Hyg^R^, G418^R^, Nat^R^)	This Study
Y243 *snf1Δ*	Y22-3 *gre3*Δ::MR *isu1*Δ::loxP-Hyg *ira2*Δ::MR *snf1Δ*::*NatMX* (Hyg^R^, Nat^R^)	This Study
Y243 *bcy1Δsnf1Δ*	Y22-3 *gre3*Δ::MR *isu1Δ*::loxP-Hyg *ira2Δ*::MR *bcy1Δ*::*KanMX snf1Δ*::*NatMX* (Hyg^R^, G418^R^, Nat^R^)	This Study
Y133 *snf1Δ SNF1* MoBY	Y133 *snf1Δ*::*Hyg* containing *SNF1* MoBY 2.0 Plasmid (Hyg^R^, G418^R^)	This Study
Y133 *snf1Δ* MoBY Control	Y133 *snf1Δ*::*Hyg* containing Empty Vector MoBY 2.0 Plasmid (Hyg^R^, G418^R^)	This Study
Y133 *mga2Δ*	Y133 *mga2Δ*::*KanMX* (G418^R^)	This Study
Y133 *MGA2* MoBY	Y133 containing *MGA2* MoBY 2.0 Plasmid (G418^R^)	This Study
Y133 *tpk1Δ*	Y133 *tpk1Δ*::*KanMX* (G418^R^)	This Study
Y133 *tpk2Δ*	Y133 *tpk2Δ*::*KanMX* (G418^R^)	This Study
Y133 *tpk3Δ*	Y133 *tpk3Δ*::*KanMX* (G418^R^)	This Study
Y133 *tpk1Δ tpk2Δ*	Y133 *tpk1Δ*::*KanMX tpk2Δ*::*Hph* (G418^R^, Hyg^R^)	This Study
Y133 *tpk1Δ tpk3Δ*	Y133 *tpk1Δ*::*KanMX tpk3Δ*::*Hph* (G418^R^, Hyg^R^)	This Study
Y133 *tpk2Δ tpk3Δ*	Y133 *tpk2Δ*::*KanMX tpk3Δ*::*Hph* (G418^R^, Hyg^R^)	This Study
Y133 *tpk2*^*as*^	Y133 *tpk1Δ*::*KanMX tpk3Δ*::*Hph tpk2*^*as*^ (G418^R^, Hyg^R^)	This Study, [59]
Y184 *bcy1Δ* p*BCY1*	Y22-3 *gre3*Δ::MR *isu1*Δ::loxP-Hyg *bcy1Δ*::*KanMX* containing p*BCY1* CEN Plasmid (Hyg^R^, G418^R^, Nat^R^)	This Study
Y184 *bcy1Δ* Empty Control	Y22-3 *gre3*Δ::MR *isu1*Δ::loxP-Hyg *bcy1Δ*::*KanMX* containing empty vector CEN Plasmid (Hyg^R^, G418^R^, Nat^R^)	This Study
Y132 *BCY1-*3' AiD	Y132 *BCY1*-3' AiD tag (3x Mini-Auxin Induced Degron Sequence-5x FLAG-*BCY1*-3' UTR-*KanMX*) (G418^R^)	This Study, [103]
Y184 *BCY1*-3' AiD	Y22-3 *gre3Δisu1Δ BCY1*-3' AiD tag (3x Mini-Auxin Induced Degron Sequence-5x FLAG-*BCY1*-3' UTR-*KanMX*) (G418^R^)	This Study, [103]
Y243 *BCY1*-3' AiD	Y36 *gre3Δisu1Δira2Δ BCY1*-3' AiD tag (3x Mini-Auxin Induced Degron Sequence-5x FLAG-*BCY1*-3' UTR-*KanMX*) (G418^R^)	This Study, [103]

Y133 *tpk1Δtpk3Δtpk2*^as^ was generated using CRISPR/Cas9-mediated genome editing. *TPK1* and *TPK3* were deleted in Y133 independently and verified by PCR. sgRNA sequence (GTGATGGATTATATCAGAAGG) that targeted the location within *TPK2* to be replaced was cloned into the pXIPHOS vector using *Not1* (GenBank accession MG897154), which contains the constitutive *RNR2* promoter driving the Cas9 gene and *NatMX* resistance gene, using gapped plasmid repair using HiFi DNA Assembly Master Mix from NEB. *tpk2*^*as*^ repair templates were generated by PCR of the whole ORF of *tpk2*^*as*^ from a strain containing mutants of the *TPK* genes that are sensitive to the ATP-analogue inhibitor 1-NM-PP1 (*TPK1* M164G, *TPK2* M147G, *TPK3* M165G) [59]. Purified repair templates were co-transformed at a 20-fold molar excess with the pXIPHOS-*TPK2* sgRNA plasmid into the Y133 *tpk1Δtpk3Δ* strain. Colonies resistant to nourseothricin were restreaked onto YPD two times to remove the plasmid (and were verified to now be sensitive to nourseothricin) and *tpk2*^*as*^ presence was verified by sequencing. Y133 *tpk1Δtpk3Δtpk2*^*as*^ was grown in xylose anaerobically for 17 hours at which point 10 μM 1-NM-PP1 or DMSO control was added to the cultures.

### Transcriptomic sample collection, library construction, and sequencing

Y22-3, Y127, and Y128 grown in YPD or YPX, with or without oxygen, were collected in biological duplicate on different days. Data from replicates were highly correlated (average R^2^ of log2(fold changes) = 0.93) and additional statistical power was incurred by analyzing across all strain data. Duplicates were used due to limitations with phosphoproteomic techniques (see below). Cultures were inoculated from a saturated aerobic sample grown in rich glucose medium (YPD), washed with the corresponding growth media, and grown for ~3 generations aerobically or anaerobically until the cultures reached mid-log phase (OD_600_ of ~0.5). Strains Y22-3 and Y127 were inoculated in rich xylose medium (YPX) at an OD_600_ of ~0.5 and incubated anaerobically for the same amount of time as the other cultures. Y22-3 and Y127 retained over 95% viability as measured by CFU/mL after 17 hours of anaerobic incubation on xylose. Growth was halted by adding 30 mL of culture to ice cold 3.75 mL 5% phenol (pH < 5)/95% ethanol solution, cultures were spun for 3 min at 3000 rpm, the decanted pellet was flash frozen in liquid nitrogen and stored at -80°C until needed. Total RNA was isolated by hot phenol lysis [105] and DNA was digested using Turbo-DNase (Life Technologies, Carlsbad, CA) for 30 min at 37°C, followed by RNA precipitation at -20°C in 2.5 M LiCl for 30 min. rRNA depletion was performed using EpiCentre Ribo-Zero Magnetic Gold Kit (Yeast) RevA kit (Illumina Inc, San Diego, CA) and purified using Agencourt RNACleanXP (Beckman Coulter, Indianapolis, IN) following manufacturers’ protocols. RNA-seq library generation was performed using the Illumina TruSeq stranded total RNA kit (Illumina) using the sample preparation guide (revision C) with minor modifications, AMPure XP bead for PCR purification (Beckman Coulter, Indianapolis, IN), and SuperScript II reverse transcriptase (Invitrogen, Carlsbad, CA) as described in the Illumina kit. Libraries were standardized to 2 μM. Cluster generation was performed using standard Cluster kits (version 3) and the Illumina Cluster station. Single-end 100-bp reads were generated using standard SBS chemistry (version 3) on an Illumina HiSeq 2000 sequencer. All raw data were deposited in the NIH GEO database under project number GSE92908.

Y133, Y133 *azf1Δ*, Y133 with the *AZF1* MoBY 2.0 plasmid, and Y133 carrying the MoBY 2.0 empty-vector control were grown in xylose -O_2_ (+/- G418 as needed), duplicate samples were collected on different days and RNA was isolated and DNA digested as described above. We focused on genes affected in multiple strains for increased statistical power. rRNA depletion was performed using EpiCentre Ribo-Zero Magnetic Gold Kit (Yeast) RevA kit (Illumina) following manufacturer’s protocols and cleaned using Qiagen RNease MinElute Cleanup kit (Qiagen, Hilden, Germany). RNA-seq library generation was performed using the EpiCentre Strand Specific ScriptSeq Kit (Illumina) as above except that Axygen AxyPrep Mag PCR Clean-up Kits for PCR purification (Axygen, Corning, NY) were used and LM-PCR was performed using 12 cycles using EpiCentre ScriptSeq Index PCR Primers (Illumina) and Epicenter Failsafe PCR Enzyme Mix (Illumina). Single-end 100-bp reads were generated using standard SBS chemistry (version 4) on an Illumina HiSeq 2500 sequencer and the two FASTQ files for each sample were combined using the “cat” command.

### RNA-seq processing and analysis

Reads for all RNA-seq experiments were processed with Trimmomatic version 0.3 [106] and mapped to the Y22-3 genome [107] using Bowtie 2 version 2.2.2 [108] with default settings. HTSeq version 0.6.0 [109] was used to calculate read counts for each gene using the Y22-3 annotation. Differential expression analysis was performed using edgeR version 3.6.8 [110] using pairwise comparisons, taking Benjamini and Hochberg [111] false discovery rate (FDR) < 0.05 as significant. Raw sequences were normalized using the reads per kilobase per million mapped reads (RPKM) method. Clustering analysis was performed using MClust version 4.4 [112] and visualized using Java TreeView (http://jtreeview.sourceforge.net) [113]. Functional enrichment analysis was performed using the FunSpec database [114,115] or a hypergeometric test using GO annotation terms (downloaded 2017-10-18) [116]. All examined targets of TFs were obtained from YeasTract [117] using only those with DNA binding evidence.

### Azf1 motif identification

We analyzed the log_2_(fold change) in expression for each strain grown anaerobically in xylose compared to anaerobically in glucose. Genes with a progressive xylose-responsive induction across the strain panel were identified if the replicate-averaged log_2_(fold-change) in Y127 was ≥ 1.5 fold higher than in Y22-3, and if the replicate-averaged log_2_(fold-change) in Y128 was also ≥ 1.5 fold higher than in Y127 (S4 Table). 21 classical hypoxic genes, those known to be involved in the hypoxic response, were selected from the literature to measure the hypoxic response (S3 Table) and for enrichment analysis to score the hypoxic response. We selected 15 of these genes with no induction in Y22-3 grown anaerobically on xylose and performed motif analysis, by extracting 1000 bp upstream of these genes and submitting to MEME [118] using the ‘any number of sequences’ model. The top motif matched the Azf1 binding site in TomTom [119]. WebLogo [120] was used to construct the final PWM logos for publication. Matches to this matrix were identified in 500bp upstream regions in the Y22-3 genome using MAST [121] with default settings. A total of 433 significant (E-value < 10) sites were identified in all intergenic regions in the genome.

### Analysis of expression in *azf1Δ* and *AZF1*-over-expressing strains

Differentially expressed genes were identified using edgeR as described above, comparing Y133 *azf1Δ* to Y133 (identifying 441 differentially expressed genes at FDR < 0.05) and comparing Y133 *AZF1* MoBY 2.0 compared to Y133 carrying the empty vector control (1,525 genes at FDR < 0.05) (S5 Table). We identified 411 genes whose expression was significantly altered (FDR < 0.05) by *AZF1* over-expression and whose replicate-averaged expression was at least 1.5X different in Y128 *versus* Y22-3 cultured anaerobically on xylose and whose expression showed the same directionality as in response to *AZF1* over-expression (S5 Table). That is, genes that showed an increase in expression when *AZF1* was over-expressed (relative to the control) also showed an increase in expression in Y128 (relative to Y22-3), and vice versa. Functional enrichment analysis was performed using the FunSpec database or hypergeometric test of GO annotation terms (downloaded 2017-10-18) [116] or compiled sets of TF targets [116].

### Label free quantitative proteomics preparation and analysis

For comparison of the Y22-3, Y127, and 128 proteomes, duplicate samples were collected from the same samples used for RNA-seq above. Duplicates were used due to limitations with phosphoproteomic techniques (see below). 35 mL of cultures were spun for 3 min at 3000 rpm, the supernatant was removed and the pellet was flash frozen in liquid nitrogen and stored at -80°C.

Label free proteomics were performed similarly to previous work [107,122]. For protein extraction and digestion, yeast cell pellets were lysed by glass bead milling (Retsch GmbH, Germany). Lysate protein concentration was measured via bicinchoninic acid protein assay (Thermo Pierce, Rockford, IL), and yeast proteins were reduced through incubation in 5mM dithiothreitol (DTT) for 45 minutes at 58°C. Free cysteines were alkylated in 15 mM iodoacetamide in the dark for 30 minutes. The alkylation was stopped with 5mM DTT. A 1 mg protein aliquot was digested overnight at room temperature in 1.5 M urea with trypsin (Promega, Madison, WI) added at a 1:50 (w/w) enzyme to protein ratio. Digestions were quenched by the addition of trifluoroacetic acid (TFA, Thermo Pierce) and were desalted over tC18 Sep-Pak cartridges (Waters, Milford, MA).

For online nanoflow liquid chromatography tandem mass spectrometry (nLC-MS/MS), reversed phase columns were packed-in house using 75 μm ID, 360 μm OD bare fused silica capillary. A nanoelectrospray tip was laser pulled (Sutter Instrument Company, Novato, CA) and packed with 1.7 μm diameter, 130 Å pore size Ethylene Bridged Hybrid C18 particles (Waters) to a length of 30–35 cm. Buffer A consisted of 0.2% formic acid and 5% DMSO in water, and Buffer B consisted of 0.2% formic acid in acetonitrile. Two μg of peptides were loaded onto the column in 95% buffer A for 12 min at 300 min^-1^. Gradient elution was performed at 300 nL min^-1^ and gradients increased linearly from 5 to 35% buffer B over 190 minutes, followed by an increase to 70% B at 215 minutes and a wash at 70% B for 5 minutes. The column was then re-equilibrated at 5% B for 20 minutes. Eluting peptide were ionized with electrospray ionization at +2 kV, and the inlet capillary temperature was held at 300°C on an ion trap-Orbitrap hybrid mass spectrometer (Orbitrap Elite, Thermo Fisher Scientific, San Jose, CA). Survey scans of peptide precursors were collected over the 300–1500 Thompson range in the Orbitrap with an automatic gain control target value of 1,000,000 (50 ms maximum injection time), followed by data-dependent ion trap MS/MS scans using collisional activation dissociation (CAD) of the 20 most intense peaks (AGC target value of 5,000 and maximum injection times of 100 ms). Precursors with charge states equal to one or unassigned were rejected.

Raw data was processed using MaxQuant version 1.4.1.2 [123], and tandem mass spectra were searched with the Andromeda search algorithm [124]. Oxidation of methionine was specified as a variable modification, while carbamidomethylation of cysteine was a set as a fixed modification. A precursor search tolerance of 20 ppm and a product mass tolerance of 0.35 Da were used for searches, and three missed cleavages were allowed for full trypsin specificity. Peptide spectral matches (PSMs) were made against a target-decoy custom database of the yeast strain was used, which was concatenated with a reversed sequence version of the forward database from McIlwain *et al*. [107]. Peptides were filtered to a 1% false discovery rate (FDR) and a 1% protein FDR was applied according to the target-decoy method. Proteins were identified using at least one peptide (razor + unique), where razor peptide is defined as a non-unique peptide assigned to the protein group with the most other peptides (Occam's razor principle). Proteins were quantified and normalized using MaxLFQ [125] with a label-free quantification (LFQ) minimum ratio count of 2. LFQ intensities were calculated using the match between runs feature, and MS/MS spectra were not required for LFQ comparisons. For quantitative comparisons, protein intensity values were log_2_ transformed prior to further analysis. All possible proteins were analyzed as long as the proteins were identified in both strains being compared, to maximize data obtained from this analysis. In total, 3,550 unique proteins were identified in across all strains and conditions. All raw mass spectrometry files and associated information about identifications are available on Chorus under Project ID 999 and Experiment ID 3007.

### Correlation between transcriptomic and proteomic differences across media conditions

The response to anaerobiosis was calculated for each strain growing either on glucose or xylose, as the log2 of mRNA or protein abundance in glucose -O_2_ / glucose +O_2_ or xylose -O_2_ / xylose +O_2_. The replicate-averaged log_2_(fold-change) in mRNA was compared to the log_2_(fold-change) in protein for each strain (Fig 1A).

### Phosphoproteomic analysis

Phosphoproteomic experiments were multiplexed using tandem mass tags (TMT) isobaric labels to quantitatively compare the phosphoproteomes of Y22-3, Y127, and Y128 yeast strains. 6-plex experiments were performed to compare the three strains grown on xylose under aerobic and anaerobic conditions. Yeast phosphoproteomes were obtained from cell pellets from the same cultures used for the label free experiments described above using the same protein extraction, proteolytic digestion, and desalting conditions. A second phosphoproteomic experiment used TMT tags to compare the phosphoproteomic profiles of Y184, Y184 *ira2Δ*, and Y184 *bcy1Δ* during anaerobic growth on xylose in duplicate, using the same collection and methods outlined above.

Following the generation of tryptic peptides, 500 μg of peptides from each condition were labeled with TMT 6-plex isobaric labels (Thermo Pierce) by re-suspending peptides in 200 μL of freshly made 200 mM triethylammonium biocarbonate (TEAB) and combining with 50 μL of the TMT labeling reagent resuspended in 100% acetonitrile. The samples were labeled for 4 hours, then ~5μg of material from each TMT channel was combined into a test mix and analyzed by LC-MS/MS to evaluate labeling efficiency and obtain optimal ratios for sample recombination. Samples were quenched with 1.6 μL of 50% hydroxylamine, then combined in equal amounts by mass, and desalted.

Combined TMT-labeled peptides were then enriched for phospho-peptides using immobilized metal affinity chromatography (IMAC) with magnetic beads (Qiagen, Valencia, CA). After equilibration with water, the magnetic beads were incubated with 40 mM EDTA (pH 8.0) for 30 minutes while shaking. This process was repeated for a total of two incubations. Next, the beads were washed four times with water and incubated with 30 mM FeCl_3_ for 30 minutes while shaking, and this was also repeated for a total of two incubations. Beads were then washed four times with 80% acetonitrile/0.15% TFA. The TMT-labeled peptides were re-suspended in 80% acetonitrile/0.15% TFA and incubated with the magnetic beads for 45 minutes with shaking. Unbound peptides were collected for protein analysis. Bound peptides were washed three times with 80% acetonitrile/0.15% TFA and eluted with 50% acetonitrile, 0.7% NH4OH. Eluted peptides were immediately acidified with 4% formic acid, frozen, and lyophilized. Enriched phospho-peptides were re-suspended in 20 μL 0.2% FA for LC-MS/MS analysis.

Online nanoflow liquid chromatography tandem mass spectrometry (nLC-MS/MS) was performed similarly as to the methods described above, including the same LC system and buffers, capillary reversed phase columns, gradient, and MS system and electrospray conditions. TMT phosphoproteomic experiments were also performed as single-shot (*i*.*e*., no fractionation) four-hour experiments. Survey scans of peptide precursors were collected over the 300–1500 Thompson range in the Orbitrap with a resolving power of 60,000 at 400 *m/z* and an automatic gain control target value of 1,000,000 (75 ms maximum injection time), followed by data-dependent MS/MS scans in the Orbitrap (resolving power 15,000 at 400 *m/z*) using higher-energy collisional dissociation (HCD, normalized collision energy of 35) of the 15 most intense peaks (AGC target value of 50,000 and maximum injection times of 200 ms). The first mass of MS/MS scans was fixed at 120 m/z, precursors were isolated with 1.8 Th isolation width, and precursors with charge states equal to one or unassigned were rejected. Dynamic exclusion windows were created around monoisotopic precursor peaks using 10 ppm windows, and the exclusion duration lasted for 40 seconds. Two technical replicate injections of each sample were performed.

Data processing for the TMT phosphoproteomic experiments used COMPASS [126]. The Open Mass Spectrometry Search Algorithm (OMSSA) [127] searches were performed against the same target-decoy yeast database used in the label free experiments described above. Searches were conducted using a 125 ppm precursor mass tolerance and a 0.02 Da product mass tolerance. A maximum of 3 missed tryptic cleavages were allowed. Fixed modifications were carbamidomethylation of cysteine residues, TMT 6-plex label on peptide N-termini, and TMT 6-plex on lysine. Variable modifications included oxidation of methionine; TMT 6-plex on tyrosine residues; phosphorylation of serine, threonine, and tyrosine residues; and neutral loss of phosphorylation on serine and threonine residues. A false discovery rate of 1% was used at the peptide and protein level. Within COMPASS, TMT quantification was performed and quantified peptides were grouped into proteins as described [127]. Phospho-peptide localization was performed using phosphoRS [128] integrated with COMPASS, using 75% as a localization probability cutoff to determine localized phospho-sites. Phospho-peptides with non-localized phospho-sites were discarded from further analysis. TMT reporter ion intensities were normalized for protein abundance and log2 transformed prior to further analysis. The PhosphoGRID database [129] was used to identify phospho-sites of known function. All raw mass spectrometry files and associated information about identifications are available on Chorus under Project ID 999 and Experiment IDs 3016 and 3166.

### Phosphoproteomic network analysis

We developed a novel network approach to infer kinases and phosphatases that mediate phosphoproteomic changes across the strain panel [49]. The method predicts co-regulated groups of phospho-peptides, called modules, and then searches a background network of protein-protein interactions to identify ‘shared interactor’ proteins that physically interact with more module constituent proteins then expected by chance. The method consists of four steps: to identify potentially co-regulated peptides, the method 1) classifies phospho-peptides according to phosphorylation profiles across strains and then 2) within each class, partitions peptides into ‘modules’ of peptides that share the same motif around the phosphorylated site (‘phospho-motif’). 3) To identify potential regulators of each module, the method identifies ‘shared interactor’ proteins that physically interact with more module constituents than expected by chance, and then 4) identifies the subset of shared interactors that are kinases and phosphatases, focusing on regulators whose known specificity matches the target module phospho-motif. These steps are described in more detail below.

#### 1) Classifying phospho-peptides

Phospho-peptides were partitioned into four classes based on the log_2_(fold-change) in phosphorylation in each strain grown in xylose -O_2_ versus xylose +O_2_. Class A contained phospho-peptides that show progressive increases or decreases in phosphorylation response (at least 1.5 fold difference in replicate-averaged log_2_ expression changes, as described above) across Y22-3, Y127, and Y128. This identified 182 phospho-peptides from 154 proteins that showed a progressive increase in response across Y22-3, Y127, and Y128 and 225 phospho-peptides from 150 proteins that showed a progressive decrease in response across the strains; these were separated into “Class A-increasing” and “Class A-decreasing” groups. Class B contained phospho-peptides with a unique hypoxic response in xylose in Y128 (at least 1.5 fold absolute difference in Y128 compared to both Y127 and Y22-3, and no significant difference between Y127 and Y22-3). This identified 108 phospho-peptides from 96 proteins that showed a larger response in Y128 and 157 phospho-peptides from 138 proteins that showed a smaller log_2_ fold-change in Y128; these were separated into “Class B-increasing” and “Class B-decreasing” groups.

#### 2) Identifying phosphorylation motifs

Peptides from each of the four classes defined above were partitioned into modules using the program *motif-X* [130,131] using the following parameters: extend from SGD yeast proteome; central character as s* or t*; motif width of 13; motif occurrences of 10; motif significance 1x10^-6^. Three total motifs were identified for Class A and five total motifs were identified for Class B. Groups of phospho-peptides containing the same motif are referred to as modules.

#### 3) Identifying shared interactor proteins

Under the assumption that co-regulated peptides interact with the same responsible regulator, we searched a background dataset of protein-protein interactions [132] to identify ‘shared interactors’ (SIs) that interact with more module constituents then expected by chance, using a custom Python script. The background network was taken from a previously compiled collection [116] of high and low-throughput protein-protein interactions or kinase-substrate interactions in *S*. *cerevisiae* [132,133] and contains 4,638 proteins and 25,682 directed and non-directed interactions. For each module, the script identifies all proteins from the background network that interact with more module constituent proteins then expected by chance (hypergeometric test), using Benjamini-Hochberg correction [111] and an FDR < 0.05 as significant. This analysis revealed 59 SIs connected to Class A modules and 90 SIs connected to Class B modules.

#### 4) Identifying candidate module regulators

We focused on the subset of SIs that are kinases with known specificity and phosphatases whose interactions with the module were primarily directed toward module constituents or were undirected. For the kinases with known specificity, we scored if the module phosphorylation motif matched the kinase motif as follows: Briefly, a position-weight matrix (PWM) was constructed for each module and compared to the PWM representing known kinase phosphorylation preferences from Mok *et al*. [134]. These PWMs were generated from a peptide phosphorylation spot array assay where the normalized, background-corrected value is provided as a weight for each amino acid at each position, which was converted to a frequency value by calculating the total of all signal intensities for all amino acids at each position and then dividing by the total sum of the intensities [135]. A pseudocount was used to prevent overfitting and to remove zeros that may occur in the Mok *et al*. PWMs [134]. These generated kinase PWMs were compared to the *motif-X* motifs via Kullback-Leibler Divergence (KLD) [119,136]. Statistical significance of matches was determined using a distribution of KLD scores generated from randomizing the within-column values and then shuffling the columns themselves 1000 times. This generated 63,000 random KLD scores per module motif. FDR was calculated as the number of random KLD scores with smaller values than the observed value. Kinases whose known specificity matched the module phosphorylation motif were retained for further consideration along with identified phosphatases. Using this approach, 6 kinases and 2 phosphatases were identified for Class A modules and 5 kinases were identified for Class B modules. Networks were visualized using Cytoscape (version 3.4.0). Network from Fig 3A is available in Cytoscape sif files (S1 File).

### Phosphoproteomic analysis across strains with and without *BCY1*

We identified phospho-peptides with a reproducible log_2_ expression difference of at least 1.5X in both biological replicates in Y184 *bcy1Δ* compared to Y184 (which mimics Y127) or in Y184 *bcy1Δ* compared to Y184 *ira2Δ* (which mimics Y128). Phospho-peptides were clustered using MClust version 4.4 [112] and visualized using Java TreeView (http://jtreeview.sourceforge.net) [113]. Functional enrichment analysis was performed with a hypergeometric test using data sets compiled of up-to-date GO annotation terms (downloaded 2017-10-18) [116], using as the background dataset the starting set of peptides used in this analysis. Phosphorylation motifs were identified as described above using *motif-X*.

### Metabolomics analysis

Metabolite data from Sato *et al*. [29] was analyzed to compare changes in Y128 xylose -O_2_ versus Y22-3 xylose -O_2_. A paired T-test was used to compare changes and those with a p-value ≤ 0.05 were considered significant.

### Inhibition of growth using hydroxyurea

Growth inhibition was performed using 400 mM hydroxyurea, added after 17 hours of anaerobic growth in xylose. Before and after growth inhibition, OD_600_ as well as sugar and ethanol concentrations were measured as above.

### PKA activity assay

Measurement of PKA activity was performed on lysed cells using the PKA Kinase Activity Assay Kit from ABCAM. Cultures were grown anaerobically in xylose for three doublings (to OD ~ 0.5), at which point 10 mL of cells were collected by centrifugation for 3 minutes at 3000 rpm, in preparation for lysis. Supernatant was removed under anaerobic conditions and the cells were resuspended in 1 mL of SB buffer (1 M sorbitol, 20 mM Tris HCl, pH 7.4) with 300 units of zymolyase (Amsbio) and 10 μL of protease inhibitor cocktail IV (Millipore). Cells were incubated for 10 minutes at 30°C anaerobically. Cells were collected by certification for 5 minutes at 350 xg and washed 1x with SB buffer under anaerobic conditions. Cells were resuspended in 750 μL HLB buffer (10 mM Tris HCl, pH 7.4, 10 mM NaCl, 3 mM MgCl_2_, 0.3% (vol/vol) NP-40, 10% (vol/vol) glycerol) with 10 μL protease inhibitor cocktail IV and incubated on ice for 10 minutes, anaerobically. Cultures were subjected to ten rounds in a Dounce homogenizer anaerobically to promote lysis. Lysis was verified using microscopy and total protein abundance was determined using a Bradford assay. 200 μL of cell lysate was removed and 50 μM H-89 was added as a PKA inhibitor and incubated for 10 minutes at 30°C anaerobically. The PKA Kinase Activity Assay Kit was performed following manufacture’s protocol, with the kinase reaction occurring under anaerobic conditions and the remaining steps (primary and secondary antibody incubation and washes) being performed aerobically. The reaction was detected using a TECAN Infinite 200 Pro with a wavelength of 450 nm. Positive (active PKA provided by ABCAM) and negative (no cells, blank) controls were used for each experimental reaction as verification of kit functionality. Relative PKA activity was calculated by subtracting the measured absorbance for each sample from the absorbance from the blank to remove background, followed by normalization to total protein abundance for each sample. Paired T-tests were used to determine significant differences among samples.

### Sugar consumption rate, ethanol production rate, and ethanol yield calculations

Sugar consumption rates and ethanol production rates were calculated as described previously [29] fitting the rate of sugar consumption or ethanol production (measured by HPLC-RID) normalized by the fitted rate of cell density change during the time of exponential growth in each strain. For strains that do not grow, the specific xylose consumption rate or ethanol production rate was found by calculating the rate of xylose consumption or ethanol production over the time of the experiment divided by the average cell density during the experimental time period for each strain. For the sake of comparison within our study, we calculated rates based on the change in cell density in the culture; there is a strong linear correlation (R^2^ = 0.98) between dry cell weight (DCW) (g/L) and OD_600_, indicating that normalizing by optical cell density is a valid approach for these strains and growth conditions. For Fig 4G and 4H, OD_600_ was converted to DCW (g/L) using a linear regression between the OD_600_ and DCW over the growth period, to enable comparisons with other studies (see S1 Table). Rates were compared with a paired T-test. Ethanol yield was found by dividing the total grams of ethanol produced by the total grams of sugar consumed over the experimental time period.

### Bcy1-AiD fermentations

Experiments were designed to mimic high-cell titer industrial fermentations. Cells were grown in YP-6% glucose or YP-3% xylose to match sugar concentrations in hydrolysate [137]. Strain Y184 Bcy1-AiD was grown aerobically in 6% glucose medium starting at an OD_600_ 0.1 or grown anaerobically in 3% xylose medium starting at OD_600_ 4.0. The tagged strain was compared to Y128, Y184 and Y184 *bcy1Δ*. OD_600_ and glucose, xylose, and ethanol were measured and rates were determined as described above. Bcy1-AiD stability was measured for each experiment using Western blot analysis as described previously [116]. Because the AiD tag contained a 3x-FLAG sequence, α-FLAG antibody (1:2500, Sigma) was used to detect Bcy1-AiD while α-Actin antibody (1:2500, Pierce) was used to detect actin as a loading control. The blot in S10 Fig is representative of biological triplicates.

## Supporting information

S1 Fig Proteome and transcriptome response to anaerobic xylose growth across the strain panel. A) Log_2_(fold change) in abundance of Frd1 and Osm1 proteins across all strains and growth conditions in response to anoxia. B) Log_2_(fold change) in abundance of *ANB1* mRNA across all strains and growth conditions in response to anoxia. C) Log_2_(fold change) in mRNA abundance of the 128 genes with a progressive increase anaerobic xylose induction, in Y22-3, Y127, and Y128 growing in glucose ±O_2_ and xylose ±O_2_.
(TIF)

S2 Fig Deletion and over-expression of *AZF1* influences growth and fermentation under anaerobic xylose conditions. A—F) OD_600_ (circles), sugar concentration (squares), and ethanol concentration (triangles) for Y133 (marker-rescued Y128) *azf1*Δ (red), Y133 *AZF1* over-expression (“OE”, blue), and Y133 wild type (“WT”) or empty-vector control (black) for different sugars and growth conditions as indicated. G) Average (n = 3) and standard deviation of sugar utilization rates from each strain during exponential growth. Asterisks indicate significant differences in sugar consumption rates as indicated (paired T-test).
(TIF)

S3 Fig *AZF1* over-expression increases xylose fermentation in a second strain background with Y128 mutations. OD_600_ (circles), xylose concentration (squares), and ethanol concentration (triangles) for CEN.PK113-5D with mutations required for xylose metabolism (*HOΔ*::*ScTAL1*-Cp*xylA-SsXYL3-loxP-isu1Δhog1Δgre3Δira2Δ* [29], Table 1) harboring the *AZF1* over-expression plasmid (purple) or empty vector control (black).
(TIF)

S4 Fig Transcriptomic analysis of *AZF1* deletion and over-expression during anaerobic xylose fermentation. A) Clustering analysis of log_2_(fold change) in mRNA for the 411 genes that show significant (FDR < 0.05) effects in response to over-expression of *AZF1* compared to controls and at least a 1.5 fold change in Y128 compared to Y22-3 grown anaerobically on xylose. Enriched functional groups (Bonferroni corrected p-value < 0.05) for genes in each cluster are listed on the right. B) Log_2_(fold change) in mRNA abundance for genes regulated by Mga2 in Y22-3, Y127, and Y128 cultured in glucose ±O_2_ or xylose ±O_2_. Asterisks indicate expression differences in each strain compared to Y22-3 (p < 0.001, paired T-test).
(TIF)

S5 Fig Relative phosphorylation differences for known and inferred PKA targets across the strains growing anaerobically in xylose. Heat map represents relative abundance of phospho-peptides across the panel. Each row represents a phospho-peptide as measured in strains (columns) grown in xylose with (left) and without oxygen (right). Data represent average phospho-peptide abundance relative to the mean abundance across all six data points, such that yellow indicates phospho-peptide abundance above the mean and blue indicates phospho-peptide abundance below the mean, according to the key. A) Shown are all phospho-peptides in Fig 3A that harbor a RxxS phospho-motif and fall into different categories described in the main text, including Class A (progressive increase/decrease) and Class B (Y128-specific response). B) Shown are 22 sites from panel A that are known PKA target sites identified in the KID database [133]. Protein name and phospho-site(s) are indicated for each row. Notably, some known PKA sites show increases in phosphorylation while others show decreases in phosphorylation in Y128 grown in xylose -O_2_.
(TIF)

S6 Fig PKA activity is required for anaerobic xylose utilization. A-C) OD_600_ (A), xylose concentration (B), and ethanol concentration (C) for Y133*tpk1Δtpk3Δtpk2*^*as*^ (blue) or Y133*tpk1Δtpk3ΔTPK2* (black) in the presence of 10 μM 1-NM-PP1 (dashed line) or DMSO control (solid line). Timing of 1-NM-PP1 or DMSO addition is indicated by a red arrow. D) Average (n = 3) and standard deviation of xylose consumption rates for individual and double *TPK* knockout strains in Y133. E) OD_600_ (circles), xylose concentration (squares), and ethanol concentration (triangles) for Y184 (Y22-3 *gre3Δ isu1Δ*) *AZF1* over-expression (“OE”, purple) or Y184 empty-vector control (black). OD_600_ measurements for Y184 *AZF1* OE highlighted in yellow. F) OD_600_ (circles), xylose concentration (squares), and ethanol concentration (triangles) for Y184 *ira2Δ* AZF1 over-expression (“OE”, purple) or Y184 *ira2*Δ empty-vector control (black). OD_600_ measurements for Y184 *ira2*Δ *AZF1* OE highlighted in yellow.
(TIF)

S7 Fig *SNF1* is required for anaerobic xylose and glucose fermentation. A-B) OD_600_ (circles), xylose concentration (squares), and ethanol concentration (triangles) for Y184 (Y22-3 *gre3Δ isu1Δ*) ±*SNF1* (A) and Y184 *bcy1Δ* ±*SNF1* (B) grown in xylose -O_2_. *SNF1+* strains are plotted in black and *snf1Δ* strains are plotted in orange. C-E) OD_600_ (circles), glucose concentration (squares), and ethanol concentration (triangles) for Y133 (marker-rescued Y128) ±*SNF1* (C), Y184 (Y22-3 *gre3Δ isu1Δ*) ±*SNF1* (D) and Y184 *bcy1Δ* ±*SNF1* (E) grown in glucose -O_2_. *SNF1+* strains are plotted in black and *snf1Δ* strains are plotted in orange. F) Average (n = 3) and standard deviation of glucose consumption rates for each strain ± *SNF1* during anaerobic growth on glucose. Asterisks indicate significant differences (paired T-test) as indicated. G-H) OD_600_ (circles), sugar concentration (squares), and ethanol concentration (triangles) in Y133 *snf1Δ* complemented with p*SNF1* Moby 2.0 plasmid [101] (black) and pEMPTY control vector [101] (aqua) for cells grown anaerobically in xylose (G) or glucose (H). The results show that Snf1 is essential for anaerobic xylose fermentation.
(TIF)

S8 Fig Deletion of *BCY1* influences anaerobic xylose fermentation. A-B) OD_600_ (circles), xylose concentration (squares), and ethanol concentration (triangles) for Y132 (marker-rescued Y127) ± *BCY1* (A) and Y184 *ira2Δ* ± *BCY1* (B) during growth in xylose -O_2_. *BCY1+* strains are in black and *bcy1Δ* strains are in green. C) Average (n = 3) and standard deviation of sugar utilization rates are shown for each strain ± *BCY1*. Asterisks indicate significant differences (paired T-test) as indicated. D) OD_600_ (circles), xylose concentration (squares), and ethanol concentration (triangles) in Y184 *bcy1Δ* complemented with p*BCY1* Moby 2.0 plasmid [101] (aqua) and pEMPTY control vector [101] (green) for cells grown anaerobically in xylose.
(TIF)

S9 Fig Inhibition of growth does not promote anaerobic xylose utilization. OD_600_ (circles) and xylose concentration (squares) for Y128 in the absence (black) and presence (green) of 400 mM hydroxyurea, added at the time point indicated by the red arrow, during anaerobic growth on xylose. Addition of hydroxyurea inhibits growth of Y128, but does not promote anaerobic xylose utilization.
(TIF)

S10 Fig Bcy1-AiD is stable in both glucose +O_2_ and xylose -O_2_. Western blot analysis of Bcy1-AiD using anti-FLAG antibody from cultures grown in glucose +O_2_ or xylose -O_2_ in Y184 with WT Bcy1 and Y184 with Bcy1-AiD. Anti-actin antibody was used as a loading control.
(TIF)

S1 Table Xylose utilization and ethanol production statistics comparing strains from this study to recently reported xylose fermentation strains in the literature [13, 15, 17, 18, 22–24, 26–28, 138–141]. (XLSX)

S2 Table Transcriptomic and proteomic counts and fold-change values for Y22-3, Y127, and Y128 in glucose ±O_2_ and xylose ±O_2_. (XLSX)

S3 Table Genes known to be involved in the hypoxic response used to examine the hypoxic response across the strain panel [40, 142–150]. (XLSX)

S4 Table The 128 genes that show a progressive increase in expression in xylose -O_2_ / xylose +O_2_. (XLSX)

S5 Table Transcriptomic analysis for Y133, Y133 *azf1Δ*, Y133 *AZF1* MoBY 2.0 plasmid (OE), and Y133 Empty MoBY Control plasmid strains in xylose -O_2_. (XLSX)

S6 Table Phosphoproteomic analysis for Y22-3, Y127, and Y128 in xylose ±O_2_. (XLSX)

S7 Table Phosphoproteomic analysis for Y184 (Y22-3 *gre3*Δ *isu1*Δ), Y184 *ira2*Δ, and Y184 *bcy1*Δ in xylose -O_2_. (XLSX)

S1 File SIF used for visualizing phosphorylation network from Fig 3A in Cytoscape. (SIF)

S1 Text Phosphoproteomics in Y184 *bcy1Δ* implicates responses involved in growth *versus* metabolism [27, 29–31, 75, 151–159]. (DOCX)
