# Supplementary figures and images for: Rewired cellular signaling coordinates sugar and hypoxic responses for anaerobic xylose fermentation in yeast
Source: PLoS Genet. 2019 Mar 11;15(3):e1008037. doi: 10.1371/journal.pgen.1008037 (PMC6428351; doi:10.1371/journal.pgen.1008037)

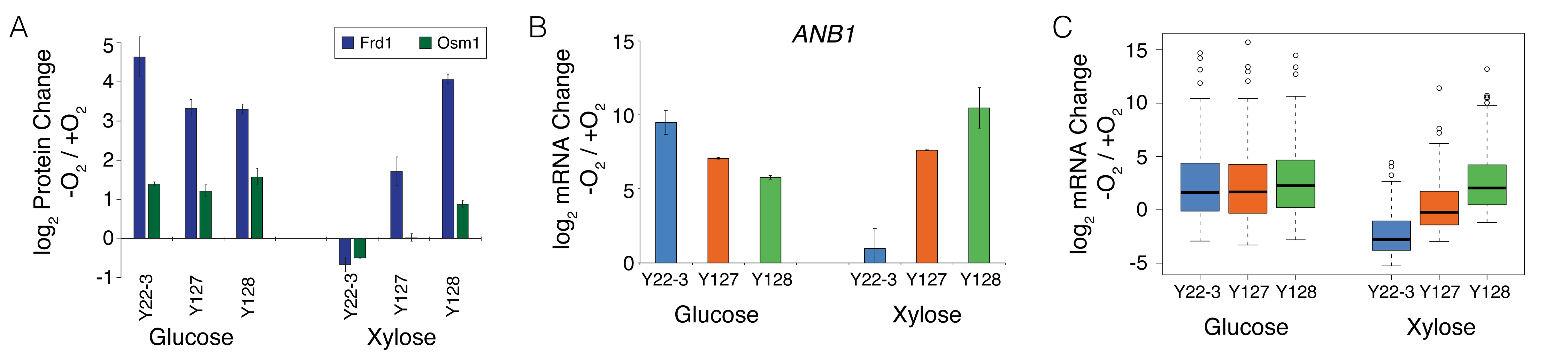

Supplement: S1 Fig — A) Log2(fold change) in abundance of Frd1 and Osm1 proteins across all strains and growth conditions in response to anoxia. B) Log2(fold change) in abundance of ANB1 mRNA across all strains and growth conditions in response to anoxia. C) Log2(fold change) in mRNA abundance of the 128 genes with a progressive increase anaerobic xylose induction, in Y22-3, Y127, and Y128 growing in glucose ±O2 and xylose ±O2. (TIF) [file pgen.1008037.s001.tif]

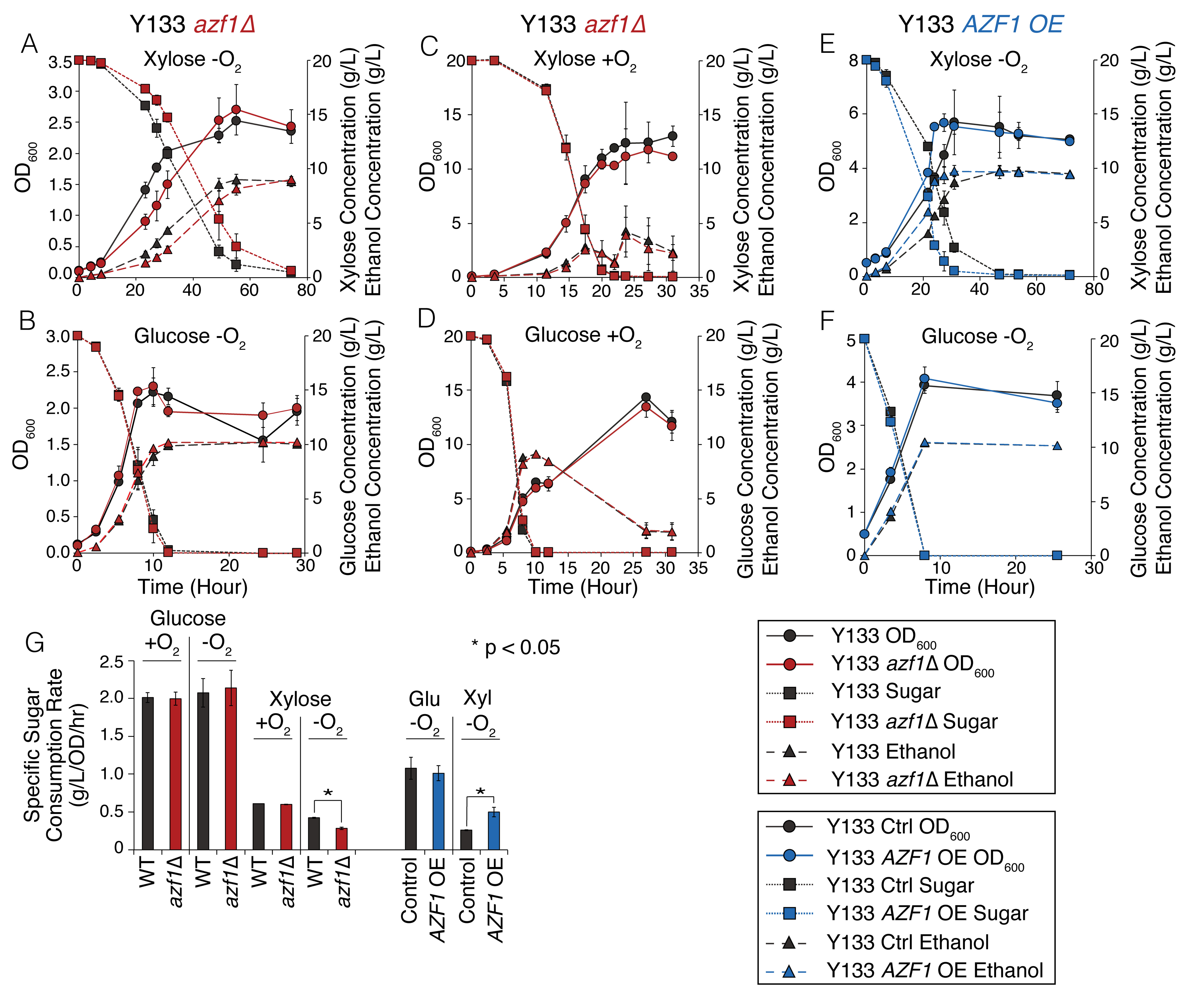

Supplement: S2 Fig — A—F) OD600 (circles), sugar concentration (squares), and ethanol concentration (triangles) for Y133 (marker-rescued Y128) azf1Δ (red), Y133 AZF1 over-expression (“OE”, blue), and Y133 wild type (“WT”) or empty-vector control (black) for different sugars and growth conditions as indicated. G) Average (n = 3) and standard deviation of sugar utilization rates from each strain during exponential growth. Asterisks indicate significant differences in sugar consumption rates as indicated (paired T-test). (TIF) [file pgen.1008037.s002.tif]

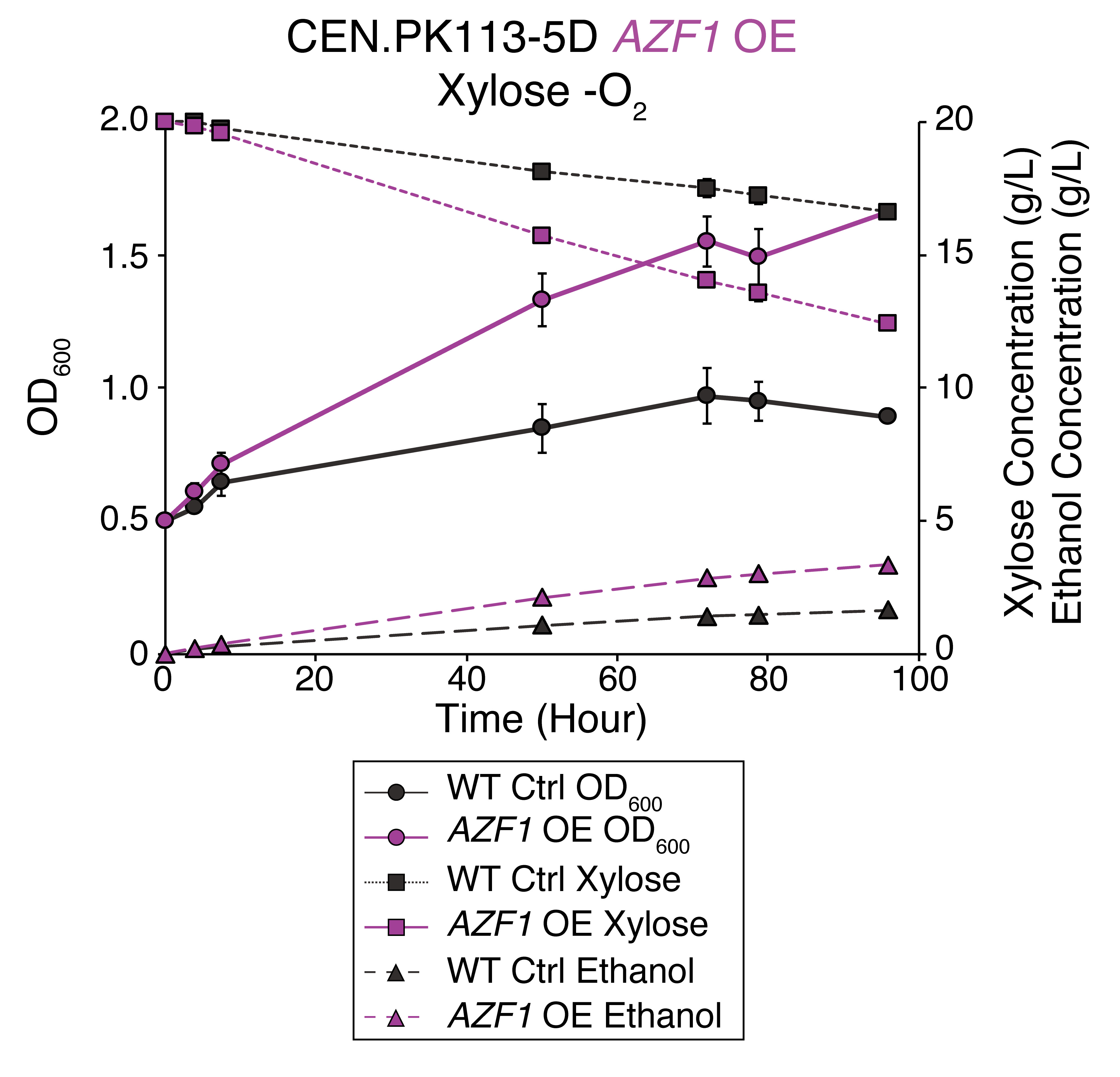

Supplement: S3 Fig — OD600 (circles), xylose concentration (squares), and ethanol concentration (triangles) for CEN.PK113-5D with mutations required for xylose metabolism (HOΔ::ScTAL1-CpxylA-SsXYL3-loxP-isu1Δhog1Δgre3Δira2Δ [29], Table 1) harboring the AZF1 over-expression plasmid (purple) or empty vector control (black). (TIF) [file pgen.1008037.s003.tif]

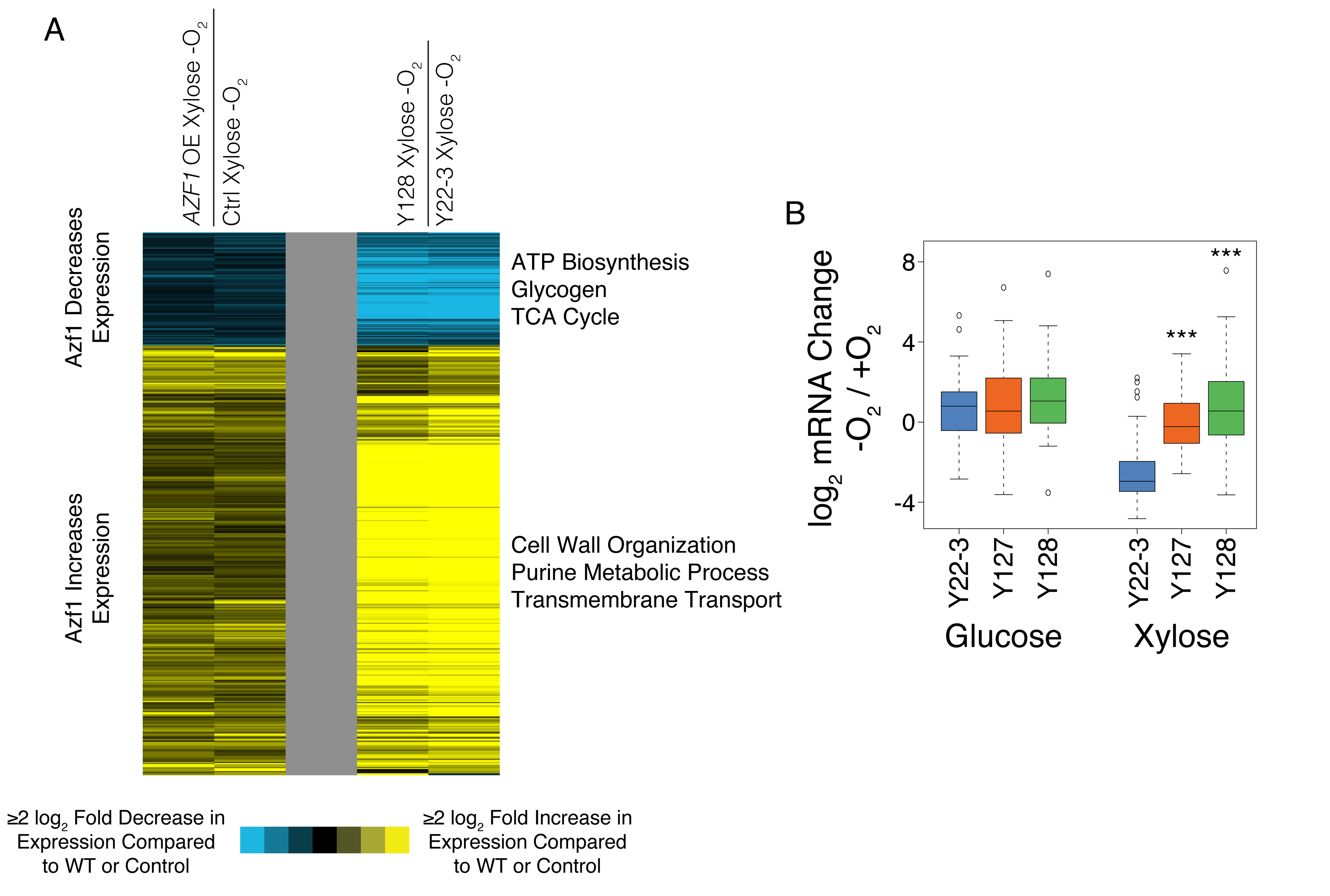

Supplement: S4 Fig — A) Clustering analysis of log2(fold change) in mRNA for the 411 genes that show significant (FDR < 0.05) effects in response to over-expression of AZF1 compared to controls and at least a 1.5 fold change in Y128 compared to Y22-3 grown anaerobically on xylose. Enriched functional groups (Bonferroni corrected p-value < 0.05) for genes in each cluster are listed on the right. B) Log2(fold change) in mRNA abundance for genes regulated by Mga2 in Y22-3, Y127, and Y128 cultured in glucose ±O2 or xylose ±O2. Asterisks indicate expression differences in each strain compared to Y22-3 (p < 0.001, paired T-test). (TIF) [file pgen.1008037.s004.tif]

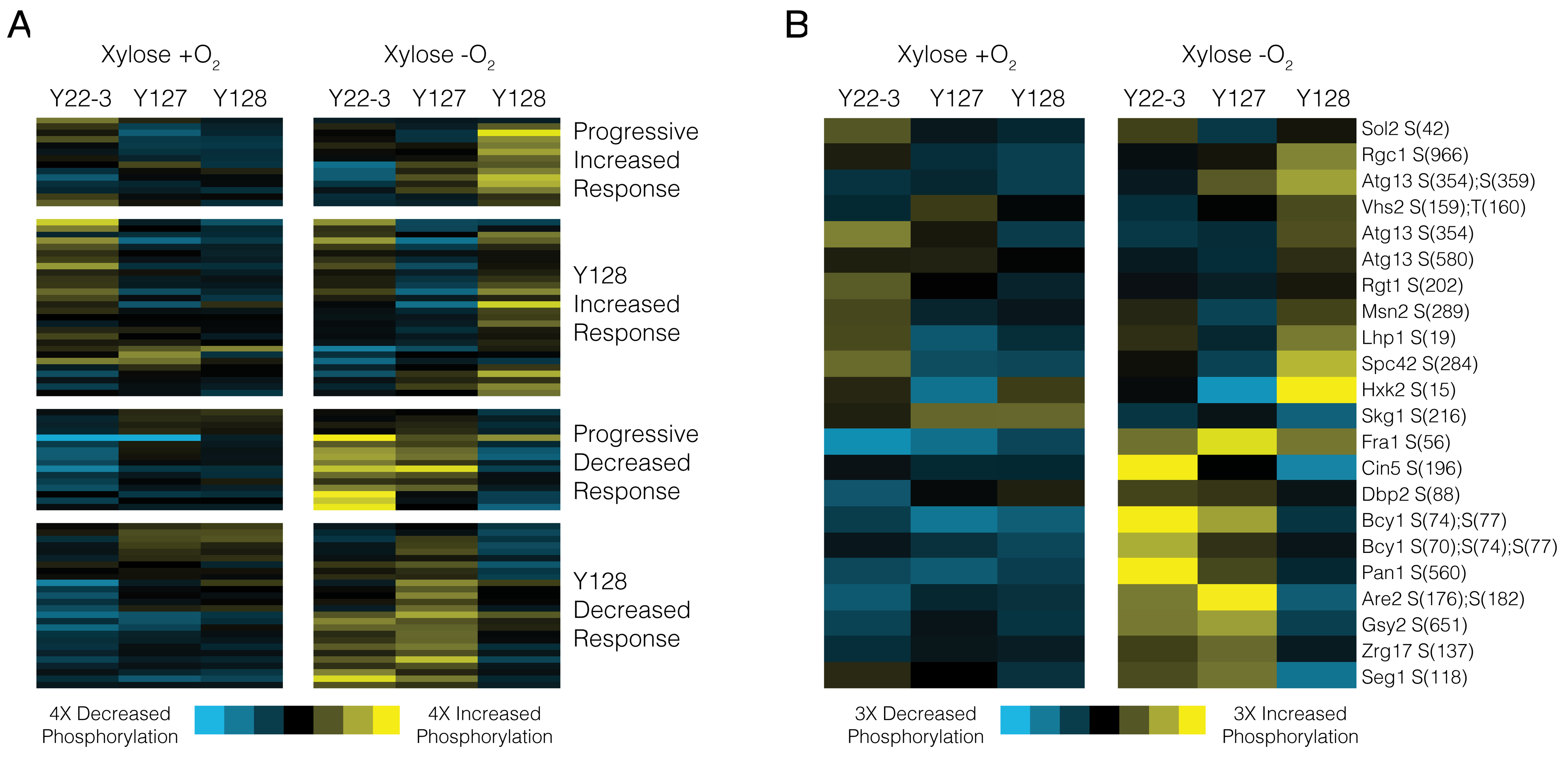

Supplement: S5 Fig — Heat map represents relative abundance of phospho-peptides across the panel. Each row represents a phospho-peptide as measured in strains (columns) grown in xylose with (left) and without oxygen (right). Data represent average phospho-peptide abundance relative to the mean abundance across all six data points, such that yellow indicates phospho-peptide abundance above the mean and blue indicates phospho-peptide abundance below the mean, according to the key. A) Shown are all phospho-peptides in Fig 3A that harbor a RxxS phospho-motif and fall into different categories described in the main text, including Class A (progressive increase/decrease) and Class B (Y128-specific response). B) Shown are 22 sites from panel A that are known PKA target sites identified in the KID database [133]. Protein name and phospho-site(s) are indicated for each row. Notably, some known PKA sites show increases in phosphorylation while others show decreases in phosphorylation in Y128 grown in xylose -O2. (TIF) [file pgen.1008037.s005.tif]

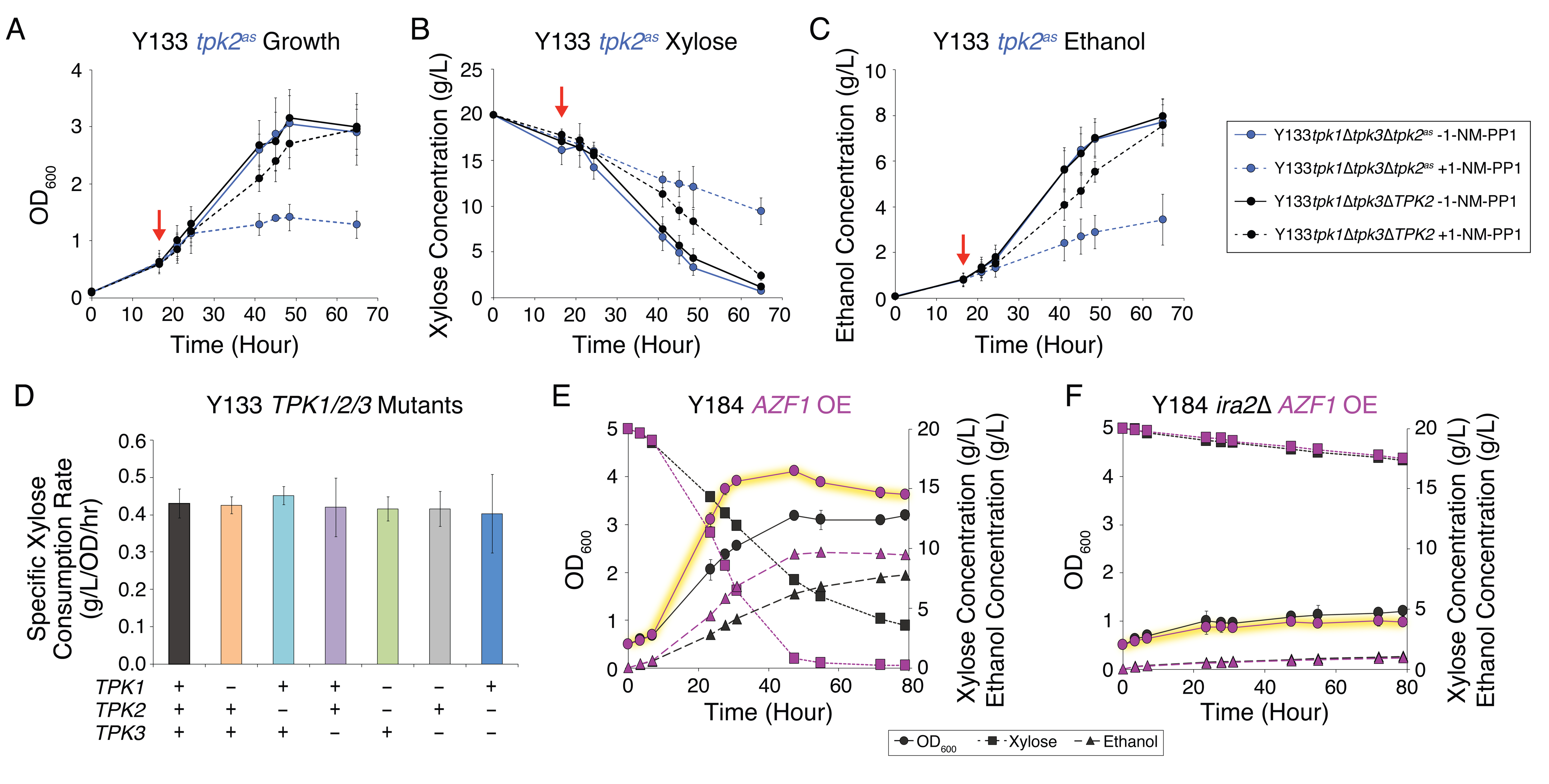

Supplement: S6 Fig — A-C) OD600 (A), xylose concentration (B), and ethanol concentration (C) for Y133tpk1Δtpk3Δtpk2as (blue) or Y133tpk1Δtpk3ΔTPK2 (black) in the presence of 10 μM 1-NM-PP1 (dashed line) or DMSO control (solid line). Timing of 1-NM-PP1 or DMSO addition is indicated by a red arrow. D) Average (n = 3) and standard deviation of xylose consumption rates for individual and double TPK knockout strains in Y133. E) OD600 (circles), xylose concentration (squares), and ethanol concentration (triangles) for Y184 (Y22-3 gre3Δ isu1Δ) AZF1 over-expression (“OE”, purple) or Y184 empty-vector control (black). OD600 measurements for Y184 AZF1 OE highlighted in yellow. F) OD600 (circles), xylose concentration (squares), and ethanol concentration (triangles) for Y184 ira2Δ AZF1 over-expression (“OE”, purple) or Y184 ira2Δ empty-vector control (black). OD600 measurements for Y184 ira2Δ AZF1 OE highlighted in yellow. (TIF) [file pgen.1008037.s006.tif]

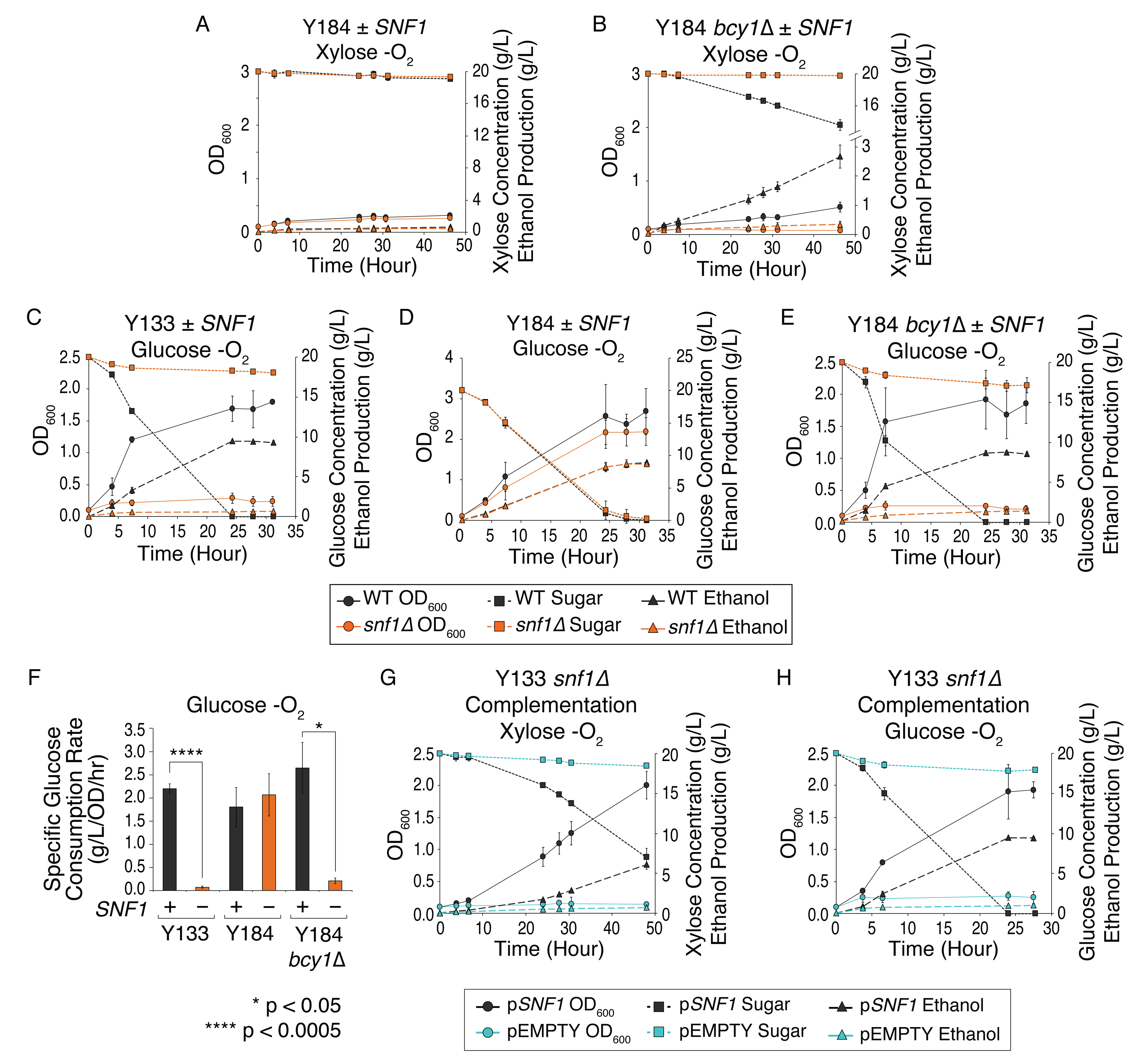

Supplement: S7 Fig — A-B) OD600 (circles), xylose concentration (squares), and ethanol concentration (triangles) for Y184 (Y22-3 gre3Δ isu1Δ) ±SNF1 (A) and Y184 bcy1Δ ±SNF1 (B) grown in xylose -O2. SNF1+ strains are plotted in black and snf1Δ strains are plotted in orange. C-E) OD600 (circles), glucose concentration (squares), and ethanol concentration (triangles) for Y133 (marker-rescued Y128) ±SNF1 (C), Y184 (Y22-3 gre3Δ isu1Δ) ±SNF1 (D) and Y184 bcy1Δ ±SNF1 (E) grown in glucose -O2. SNF1+ strains are plotted in black and snf1Δ strains are plotted in orange. F) Average (n = 3) and standard deviation of glucose consumption rates for each strain ± SNF1 during anaerobic growth on glucose. Asterisks indicate significant differences (paired T-test) as indicated. G-H) OD600 (circles), sugar concentration (squares), and ethanol concentration (triangles) in Y133 snf1Δ complemented with pSNF1 Moby 2.0 plasmid [101] (black) and pEMPTY control vector [101] (aqua) for cells grown anaerobically in xylose (G) or glucose (H). The results show that Snf1 is essential for anaerobic xylose fermentation. (TIF) [file pgen.1008037.s007.tif]

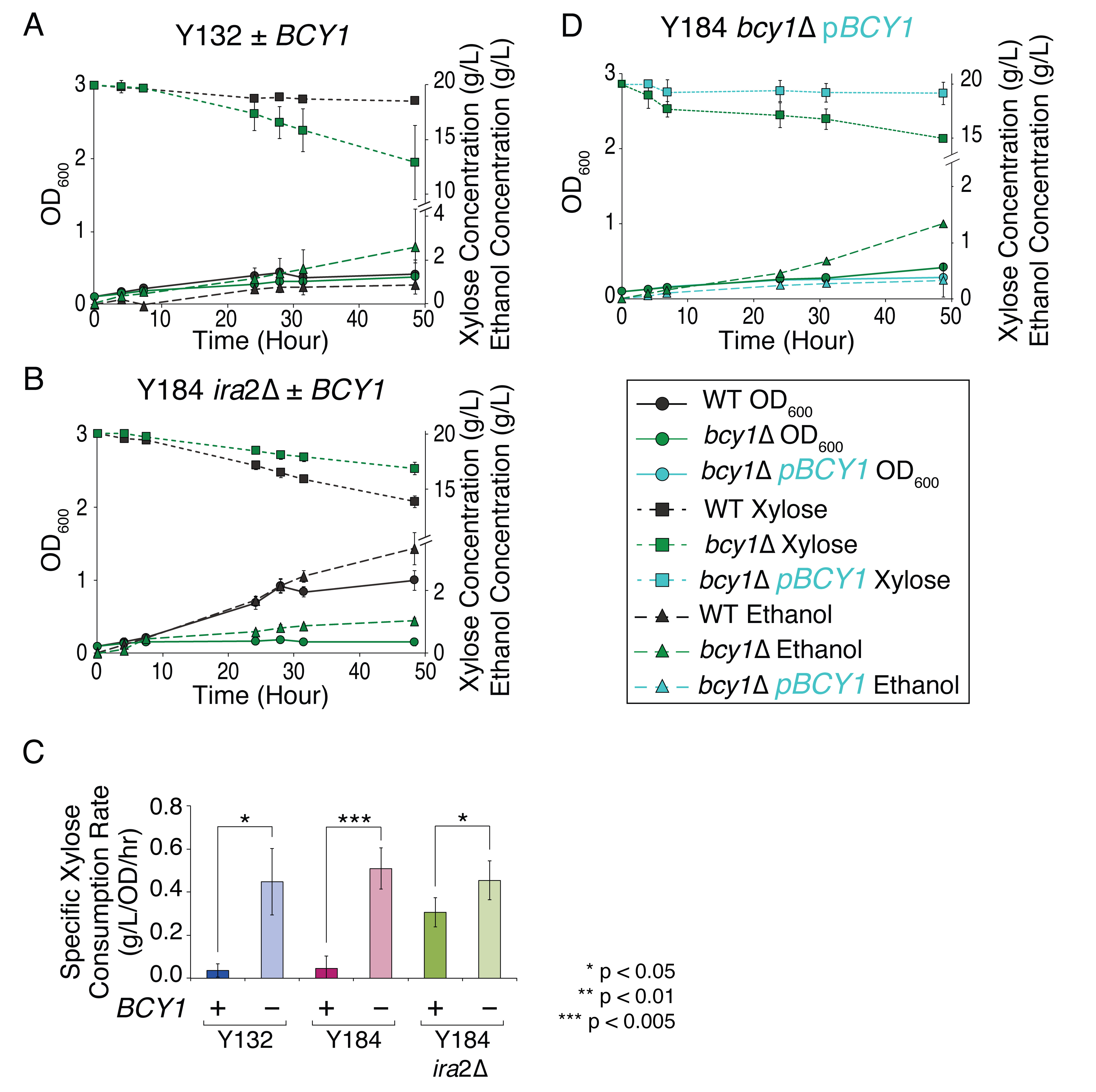

Supplement: S8 Fig — A-B) OD600 (circles), xylose concentration (squares), and ethanol concentration (triangles) for Y132 (marker-rescued Y127) ± BCY1 (A) and Y184 ira2Δ ± BCY1 (B) during growth in xylose -O2. BCY1+ strains are in black and bcy1Δ strains are in green. C) Average (n = 3) and standard deviation of sugar utilization rates are shown for each strain ± BCY1. Asterisks indicate significant differences (paired T-test) as indicated. D) OD600 (circles), xylose concentration (squares), and ethanol concentration (triangles) in Y184 bcy1Δ complemented with pBCY1 Moby 2.0 plasmid [101] (aqua) and pEMPTY control vector [101] (green) for cells grown anaerobically in xylose. (TIF) [file pgen.1008037.s008.tif]

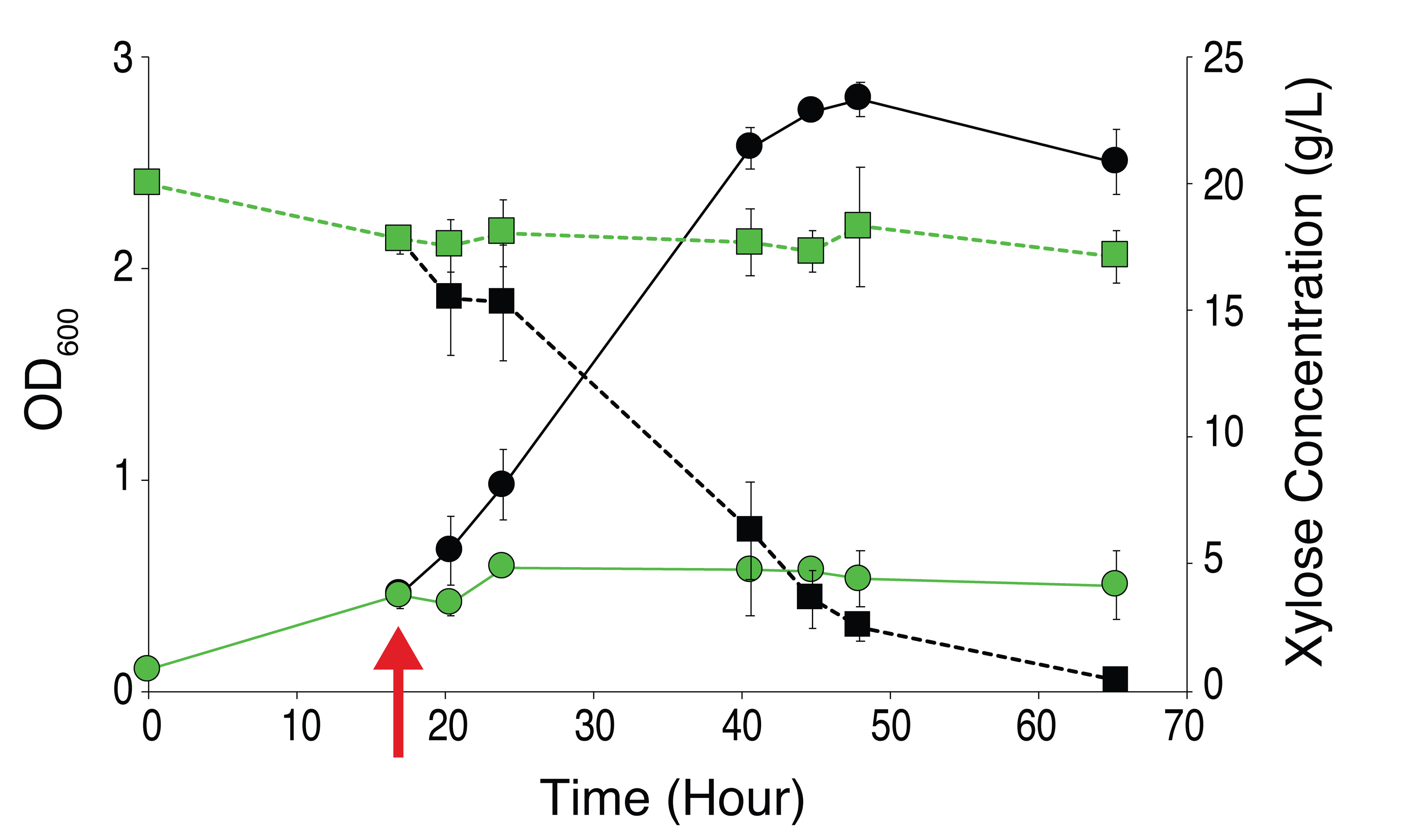

Supplement: S9 Fig — OD600 (circles) and xylose concentration (squares) for Y128 in the absence (black) and presence (green) of 400 mM hydroxyurea, added at the time point indicated by the red arrow, during anaerobic growth on xylose. Addition of hydroxyurea inhibits growth of Y128, but does not promote anaerobic xylose utilization. (TIF) [file pgen.1008037.s009.tif]

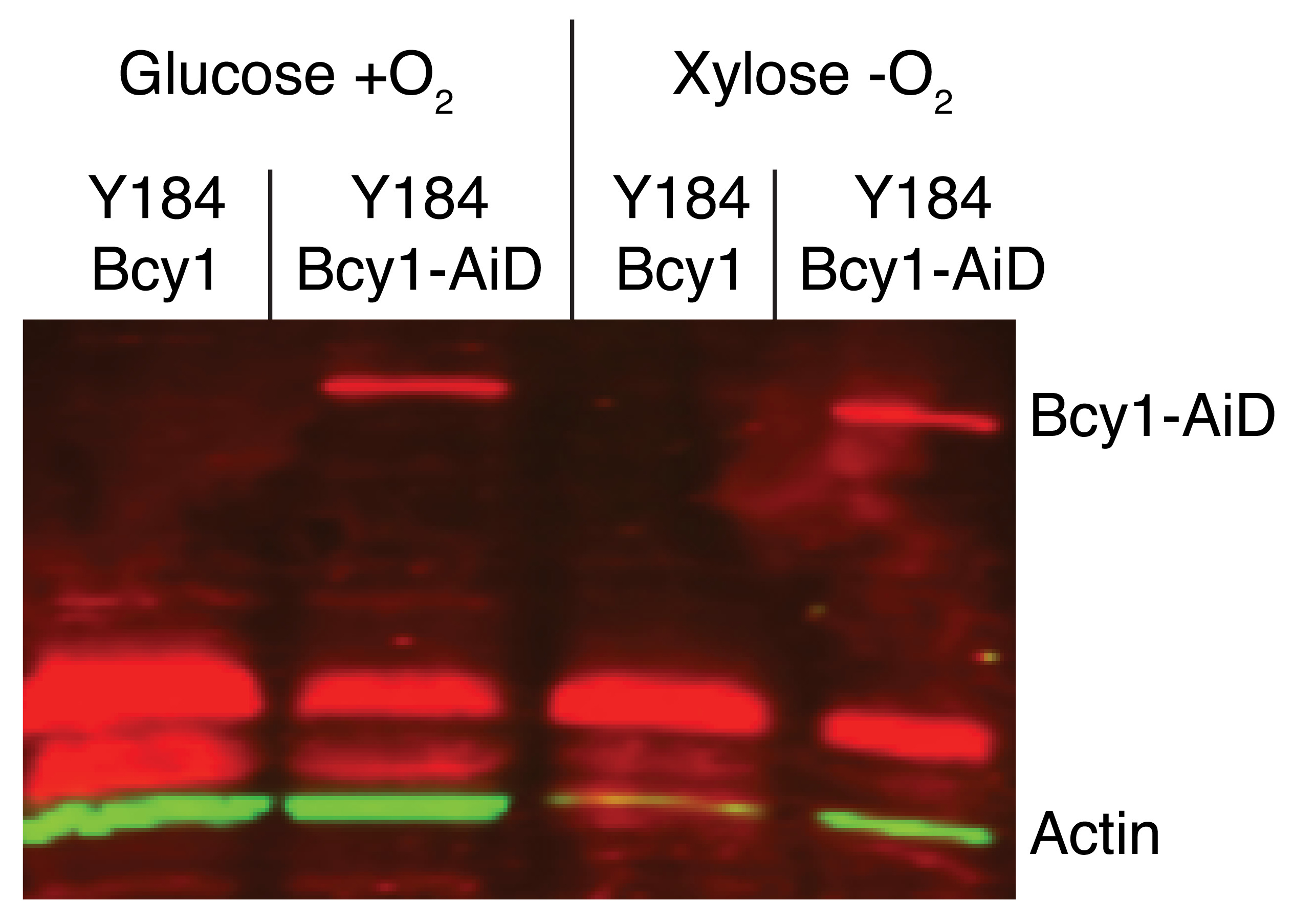

Supplement: S10 Fig — Western blot analysis of Bcy1-AiD using anti-FLAG antibody from cultures grown in glucose +O2 or xylose -O2 in Y184 with WT Bcy1 and Y184 with Bcy1-AiD. Anti-actin antibody was used as a loading control. (TIF) [file pgen.1008037.s010.tif]
