# Supplementary material for: Rewired cellular signaling coordinates sugar and hypoxic responses for anaerobic xylose fermentation in yeast
Source: PLoS Genet. 2019 Mar 11;15(3):e1008037. doi: 10.1371/journal.pgen.1008037 (PMC6428351; doi:10.1371/journal.pgen.1008037)
Supplement: S1 Text — (DOCX) [file pgen.1008037.s019.docx]

SI Text

**Phosphoproteomics in Y184 *bcy1∆* implicates responses involved in growth *versus* metabolism**

We compared phosphoproteomic data among three strains cultured anaerobically on xylose: Y184 (Y22-3 *gre3∆ isu1∆*) that can neither grow on nor metabolize xylose, Y184 *ira2∆* (Y22-3 *gre3∆isu1∆ira2∆*) that both grows on and metabolizes xylose, and Y184 *bcy1∆* (Y22-3 *gre3∆ isu1∆bcy1∆*) that does not grow on but metabolizes xylose. In total, 541 phospho-peptides showed a ≥1.5 fold difference in abundance between Y184 *bcy1∆* and Y184 or Y184 *ira2∆,* in both biological replicates (Table S7). These peptides were classified into three groups: Class I peptides are those with a difference in phosphorylation level in Y184 *bcy1∆* cultured anaerobically on xylose relative to both Y184 and Y184 *ira2∆* cultured under those conditions. Class II peptides are those with differences between the Y184 *bcy1∆* strain compared to Y184 *ira2∆* only – Class II peptides therefore represent those where the *bcy1∆* strain was more similar to Y184, neither of which grows anaerobically on xylose. Class III peptides are those whose phosphorylation was reproducibly different only in the *bcy1∆* strain compared to Y184, revealing that the *bcy1∆* strain behaved more like the Y184 *ira2∆* strain, which can also metabolize xylose (Fig. 4B-4D). We examined each cluster using enrichment and network analysis (see Materials and Methods), under the hypotheses that Class II phospho-peptides may relate to the growth defect of Y127 and Y184 *bcy1∆* whereas Class III phospho-peptides may be those associated with xylose metabolism, since both Y128 and Y184 *bcy1∆* can metabolize the sugar under these conditions.

There were 188 phosphorylation events in Class I, unique to or amplified in the in *bcy1∆* strain compared to both Y184 and Y184 *ira2∆*. These included 34 phospho-peptides (in 28 proteins) that showed increased phosphorylation in *bcy1∆* and 154 phospho-peptides (mapping to 111 proteins) with a *bcy1∆-*specific decrease (Fig. 4B). Both groups included proteins related to the stress response and glycolysis. Interestingly, the *bcy1∆* strain cultured anaerobically on xylose showed increased phosphorylation of serine 248 (S248) of Pbs2, the MAPKKK that activates the Hog1 kinase; in contrast, the strain showed reduced phosphorylation of T174 on Hog1, whose phosphorylation normally activates the kinase. This is especially intriguing because Hog1 inactivation in Y127 and other strains enhances xylose consumption [27,29]. Our data suggest that *BCY1* deletion serves to down-regulate Hog1 signaling without mutation of the gene. We previously proposed that Hog1 activity during glucose starvation may reduce growth-promoting processes, and thus deleting *HOG1* (or down-regulating its activity) may enable xylose fermentation without the corresponding limitation of growth related processes [27,29].

Multiple hexokinases were affected uniquely in the *bcy1∆* strain: Glk1 and Hxk1 both showed increased phosphorylation in the strain, whereas the main hexokinase, Hxk2, showed decreased phosphorylation on many sites (several shared with Y184 *ira2∆*). These included reduced phosphorylation on S158 that is normally autophosphorylated as a feedback mechanism to inactivate the enzyme [151], and S15 [75] whose phosphorylation normally increases activity. Hxk2 is an interesting enzyme, because it acts both in glycolysis and as a regulator of nuclear transcription via the Mig1 repressor [152,153]. Decreased phosphorylation at these sites may have broader effects here: deletion of *HXK2* results in constitutive expression of Snf1 targets to enable growth on non-glucose fermentable carbon sources [154]. Our data raise the possibility that hexokinase activity is decreased to affect how the cell senses and/or responds to glucose availability.

We next analyzed phosphoproteomic changes in Class II, where the *bcy1∆* strain (which metabolizes xylose but cannot grow on it) is more similar to Y184 that can neither grow on nor metabolize xylose (Fig. 4C). Our hypothesis is that phosphorylation of peptides in this category may be related to the shared inability of these strains to grow anaerobically on xylose. There were 22 phospho-sites (in 18 proteins) with an increase in phosphorylation in Y184 *bcy1∆* compared to Y184 *ira2∆*. Although there was no significant enrichment, there were several interesting proteins including transketolase (Tkl1) involved in pentose phosphate/xylose metabolism and intriguingly Rnr2, the main deoxyribonucleotide-diphosphate reductase critical for nucleotide biosynthesis and thus growth. Conversely, 28 phospho-sites (in 24 proteins) showed decreased in phosphorylation in the *bcy1∆* strain compared to Y184 *ira2∆*. These included several proteins involved in ribosome biogenesis (Alb1 and Zuo1), mRNA transport (Nup60), as well as Mga2 discussed in the main text. It is possible that one or more of these phosphorylation differences inhibit growth in the *bcy1∆* strain but enable continued xylose fermentation.

In contrast, Class III phosphorylation events were similar between Y184 *bcy1∆* and Y184 *ira2∆* strains*,* but distinct from Y184 that cannot metabolize xylose (Fig. 4D). The 51 phospho-sites (in 38 proteins) that displayed an increase in phosphorylation in the *bcy1∆* strain compared to Y184 were enriched for stress response proteins, including the Yak1 kinase that is antagonistic to PKA signaling and activated during times of stress [30,31]. Since PKA activity is known to suppress the stress response [155], this signature likely reflects PKA-dependent suppression of stress defense. Also included in the group of peptides with higher phosphorylation in xylose-fermenting strains is Cdc25, the guanine-nucleotide exchange factor for RAS and a known PKA target [156,157]. Increased phosphorylation on Cdc25 site S135 is thought to increase its activity, which would promote RAS-dependent signaling and PKA activity [158]. The increased phosphorylation of Cdc25 S135 is consistent with the notion that RAS/PKA activity is up-regulated by *IRA2* or *BCY1* deletion to promote increased xylose flux. The 262 phospho-sites (in 165 proteins) that showed a decrease in phosphorylation compared to Y184 were enriched for proteins involved in the regulation of cell shape, cytoskeleton, and bud site selection. Interestingly, network analysis revealed that Pkh1 (involved endocytosis control), Yck2 (involved in morphogenesis, trafficking, and glucose response), Akl1 (endocytosis and cytoskeleton organization), and Ark1 (regulation of actin cytoskeleton) kinases all showed more interactions with the proteins whose phosphorylation decreased in this class, compared to what is expected by chance. Indeed, many of the 165 proteins to which the affected peptides mapped are involved in cytoskeleton regulation. Several other sites with decreased phosphorylation in the *bcy1∆* and Y184 *ira2∆* strains have known functions related to cell cycle progression, including histone Hta1 on S129 and Cdc3 on site S503 [159]. Decreased phosphorylation of Pah1 and Cho1 involved in lipid biogenesis is also predicted to reduce activity of the enzymes. It is intriguing that so many of these regulators are linked to growth, morphology, and cell-cycle progression; however, their phosphorylation patterns are shared between the growing Y184 *ira2∆* and the arrested Y184 *bcy1∆.* One possibility is that these phosphorylation events are a unique response in the Y184 reference strain in response to its inability to grow, for reasons that are distinct than in Y184 *bcy1∆.*
